# FeatureSelect: a software for feature selection based on machine learning approaches

**DOI:** 10.1186/s12859-019-2754-0

**Published:** 2019-04-03

**Authors:** Yosef Masoudi-Sobhanzadeh, Habib Motieghader, Ali Masoudi-Nejad

**Affiliations:** Laboratory of system Biology and Bioinformatics, Institute of Biochemistry and Biophysics, University of Tehran, Tehran, Iran

**Keywords:** Feature selection, Gene selection, Machine learning, Classification, Regression

## Abstract

**Background:**

Feature selection, as a preprocessing stage, is a challenging problem in various sciences such as biology, engineering, computer science, and other fields. For this purpose, some studies have introduced tools and softwares such as WEKA. Meanwhile, these tools or softwares are based on filter methods which have lower performance relative to wrapper methods. In this paper, we address this limitation and introduce a software application called FeatureSelect. In addition to filter methods, FeatureSelect consists of optimisation algorithms and three types of learners. It provides a user-friendly and straightforward method of feature selection for use in any kind of research, and can easily be applied to any type of balanced and unbalanced data based on several score functions like accuracy, sensitivity, specificity, etc.

**Results:**

In addition to our previously introduced optimisation algorithm (WCC), a total of 10 efficient, well-known and recently developed algorithms have been implemented in FeatureSelect. We applied our software to a range of different datasets and evaluated the performance of its algorithms. Acquired results show that the performances of algorithms are varying on different datasets, but WCC, LCA, FOA, and LA are suitable than others in the overall state. The results also show that wrapper methods are better than filter methods.

**Conclusions:**

FeatureSelect is a feature or gene selection software application which is based on wrapper methods. Furthermore, it includes some popular filter methods and generates various comparison diagrams and statistical measurements. It is available from GitHub (https://github.com/LBBSoft/FeatureSelect) and is free open source software under an MIT license.

**Electronic supplementary material:**

The online version of this article (10.1186/s12859-019-2754-0) contains supplementary material, which is available to authorized users.

## Background

Data preprocessing is an essential component of many classification and regression problems. Some data have an identical effect, some have a misleading effect and others have no effect on classification or regression problems, and the selection of an optimal and minimum size for features can therefore be useful [1]. A classification or regression problem will involve a high time complexity and low performance when a large number of features is used, but will have a low time complexity and high performance for a minimum size and the most effective features. The selection of an optimal set of features with which a classifier or a model can achieve its maximum performance is an nondeterministic polynomial (NP) problem [2]. Meta-heuristic and heuristic approaches can be applied to NP problems. Optimisation algorithms, which are a type of meta-heuristic algorithm, are usually more efficient than other meta-heuristic algorithms. After selecting an optimal subset of features, a classifier can properly classify the data, or a regression model can be constructed to estimate the relationships between variables. A classifier or a regression model can be created using three methods [3]: (i) a supervised method, in which a learner is aware of data labels; (ii) an unsupervised method, in which a learner is unaware of data labels and tries to find the relationship between data; and (iii) a semi-supervised method in which labels of some data are determined whereas others are not specified. In this method, a learner is usually trained using the both labeled and unlabeled samples. This paper introduces a software application named FeatureSelect in which three types of learner are available in: 1- SVM: A support vector machine (SVM) is one possible supervised learning method that can be applied to classification and regression problems. The aim of an SVM is to determine a line that divides two groups with the greatest margin of confidence [4]. 2- ANN: Like SVM, an artificial neural network (ANN) is a supervised learner and tries to find relation between inputs and outputs. 3- DT: Decision tree (DT) is one of the other supervised learners which can be employed for machine learning applications. FeatureSelect comprises two steps: (i) it selects an optimal subset of features using optimisation algorithms; and (ii) it uses a learner (SVM, ANN and DT) to create a classification or a regression model. After each run, FeatureSelect calculates the required statistical results for regression and classification problems, including sensitivity, fall-out, precision, convergence and stability diagrams for error, accuracy and classification, standard deviation, confidence interval and many other essential statistical results. FeatureSelect is straightforward to use and can be applied within many different fields.

Feature extraction and selection are two main steps in machine learning applications. In feature extraction, some attributes of the existing data, intended to be informative, are extracted. As an instance, we can point out some biologically related works such as Pse-in-One [5] and ProtrWeb [6] which enable users to acquire some features from biological sequences like DNA, RNA, or protein. However, all of the derived features are not constructive in process of learning a machine. Therefore, feature selection methods which are used in various fields such as drug design, disease classification, image processing, text mining, handwriting recognition, spoken word recognition, social networks, and many others, are essential. We divide related works into five categories: (i) filter-based; (ii) wrapper-based; (iii) embedded-based; (iv) online-based; (v) and hybrid-based. Some of the more recently proposed methods and algorithms based on mentioned categories are described below.

### (i) Filter-based

Because filter methods, which does not use a learning method and only considers the relevance between features, have low time complexity; many of researchers focused on these methods. In one of related works, a filter-based method has been introduced for use in online stream feature selection applications. This method has acceptable stability and scalability, and can also be used in offline feature selection applications. However, filter feature selection methods may ignore certain informative features [7]. In some cases, data are unbalanced; in other words, they are in a state of skewness. Feature selection for linear data types has also been studied, in a work that provides a framework and selects features with maximum relevance and minimum redundancy. This framework has been compared with state-of-the-art algorithms, and has been applied to nonlinear data [8].

### (ii) wrapper-based

These methods evaluate usefulness of selected features using learner’s performance [9]. In a separate study, a feature selection method was proposed in which both unbalanced and balanced data can be classified, based on a genetic algorithm. However, it has been proved that other optimisation algorithms can be more efficient than the genetic algorithm [10]. Feature selection methods not only improve the performance of the model but also facilitate the analysis of the results. One study examines the use of SVMs in multiclass problems. This work proposes an iterative method based on a features list combination that ranks the features and examines only features list combination strategies. The results show that a one-by-one strategy is better than the other strategies examined, for real-world datasets [11].

### (iii) embedded-based

Embedded methods select features when a model is made. For example, the methods which select features using decision tree are placed in this category. One of the embedded methods investigates feature selection with regard to the relationships between features and labels and the relationships among features. The method proposed in this study was applied to customer classification data, and the proposed algorithm was trained using deterministic score models such as the Fisher score, the Laplacian score, and two semi-supervised algorithms. This method can also be trained using fewer samples, and stochastic algorithms can improve the performance of the algorithm [12]. As mentioned above, feature selection is currently a topic of great research interest in the field of machine learning. The nature of the features and the degree to which they can be distinguished are not considered. The concept has been introduced and examined for benchmark datasets by Liu, et al. This method is appropriate for multimodal data types [13].

### (iv) online-based

These methods select features using online user tips. In a related work, a feature cluster taxonomy feature selection (FCTFS) method has been introduced. The main goal of FCTFS is the selection of features based on a user-guided mode. The accuracy of this method is lower than that of the other methods [14]. In a separate study, an online feature selection method based on the dependency on the k nearest neighbours (k-OFSD) has been proposed, and this is suitable for high-dimensional datasets. The main motivation for the abovementioned work is the selection of features with a higher ability to separate those for which the performance has been examined using unbalanced data [15]. A library of online feature selection (LOFS) has also been developed using the state-of-art algorithms, for use with MATLAB and OCTAVE. Since the performance of LOFS has not been examined for a range of datasets, its performance has not been investigated [16].

### (v) Hybrid-based

These methods are combination of four above categories. For example, some related works use two-step feature selection methods [17, 18]. In these methods, a number of features are reduced by the first method, and the second method is then used for further reduction [19]. While some works focus on only one of these categories, a hybrid two-step feature selection method, which combines the filter and wrapper methods, has been proposed for multi-word recognition. It is possible to remove the most discriminative features in the filter method, so that this method is solely dependent on the filter stage [20]. DNA microarray datasets usually have a large size and a large number of features, and feature selection can reduce the size of this dataset, allowing a classifier to properly classify the data. For this purpose, a new hybrid algorithm has been suggested that combines the maximisation of mutual information with a genetic algorithm. Although the proposed method increases the accuracy, it appears that other state-of-the-art optimisation algorithms can improve accuracy to a greater extent than the genetic algorithm [21–23]. Defining a framework for the relationship between Bayesian error and mutual information [24], and proposing a discrete optimisation algorithm based on opinion formation [25] are other hybrid methods.

**Fig. 1:**
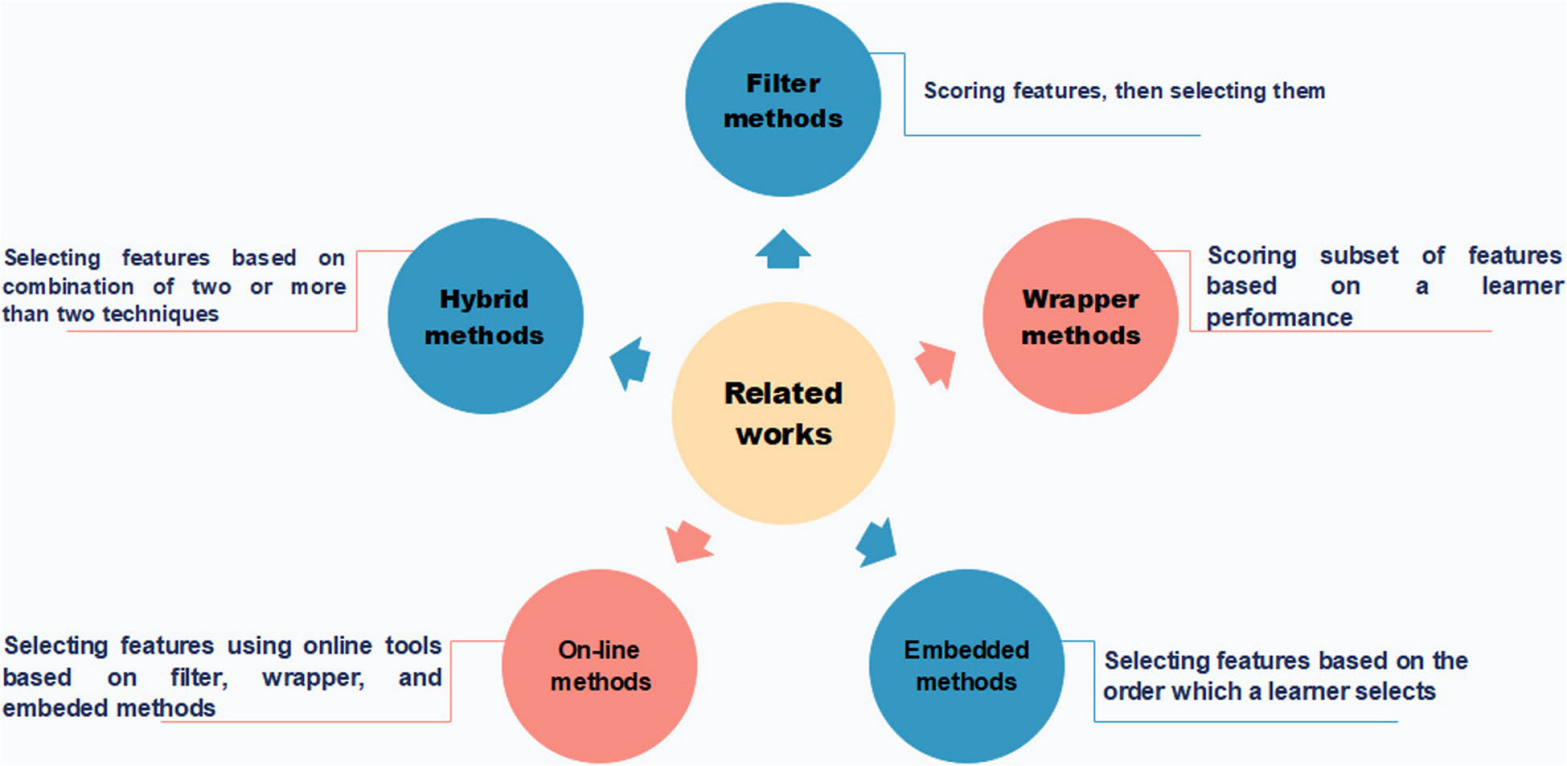
Classification of the related works. They have been categorized into five classes, including: (i) Filter method which scores features and then selects them. (ii) Wrapper method which scores a subset of features based on a learner performance. (iii) Embedded method which selects features based on the order that a learner selects them. (iv) Online method which is based online tools. (V) Hybrid method which combines different methods in order to acquire better results

Other recent topics of study include review studies or feature selection in special area. A comprehensive and extensive review of over various relevant works was carried out by researchers. The scope, applications and restrictions of these works were also investigated [26–28]. Some other related works are as below: Unsupervised feature selection methods [29–31], feature selection using a variable number of features [32], connecting data characteristics using feature selection [33–36], a new method for feature selection using feature self-representation and a low-rank representation [36], integrating feature selection algorithms [37], financial distress prediction using feature selection [38], and feature selection based on a Morisita estimator for regression problems [39]. Figure 1 summarizes and describes the above categories in a graphical manner.

FeatureSelect is placed in the filter, wrapper, and hybrid categories. In the wrapper method, FeatureSelect scores a subset of features instead of scoring features separately. To this end, the optimization algorithms select a subset of features. Next, the selected subset is scored by a learner. In addition to the wrapper method, FeatureSelect includes 5 filter methods which can score features using Laplacian [40], entropy [41], Fisher [42], Pearson-correlation [43], and mutual information [44] scores. After scoring, it selects features based on their scores. Furthermore, this software can be used in a hybrid manner. For example, a user can reduce the number of features using the filter method. Then, the reduced set can be used as input for the wrapper method in order to enhance the performance.

## Implementation

Data classification is a subject that has attracted a great deal of research interest in the domain of machine learning applications. An SVM can be used to construct a hyperplane between groups of data, and this approach can be applied to linear or multiclass classification and regression problems. The hyperplane has a suitable separation ability if it can maintain the largest distance from the points in either class; in other words, the high separation ability of the hyperplane is determined by a functional margin. The higher the value of a functional margin, the lower is the error in the value [45]. Several modified versions of an SVM have also been proposed [46].

Because SVM is a popular classifier in the area of machine learning, Chang and Lin have designed a library for support vector machine named LIBSVM [47], which has several important properties, as follows:

- It can easily be linked to different programing languages such as MATLAB, Java, Phyton, LISP, CLISP, WEKA, R, C#, PHP, Haskell, Perl and Ruby;
- Various SVM formulations and kernels are available;
- It provides a weighted SVM for unbalanced data;
- Cross-validation can be applied to the model selection.

In addition to SVM, ANN and DT are also available as learners in FeatureSelect. In the implementation of FeatureSelect, ANN has been implemented whereas SVM and DT have been added to it as a library. ANN, which includes some hidden layers and some neurons in them and can be applied to both classification and regression problems, has been inspired by neural system of living organisms [48]. Like SVM and ANN, DT can also be used for both classification and regression issues. DT operates based on tree-like graph model and develops a tree step by step by adding new constraints which lead to desired consequences [49].

**Fig. 2:**
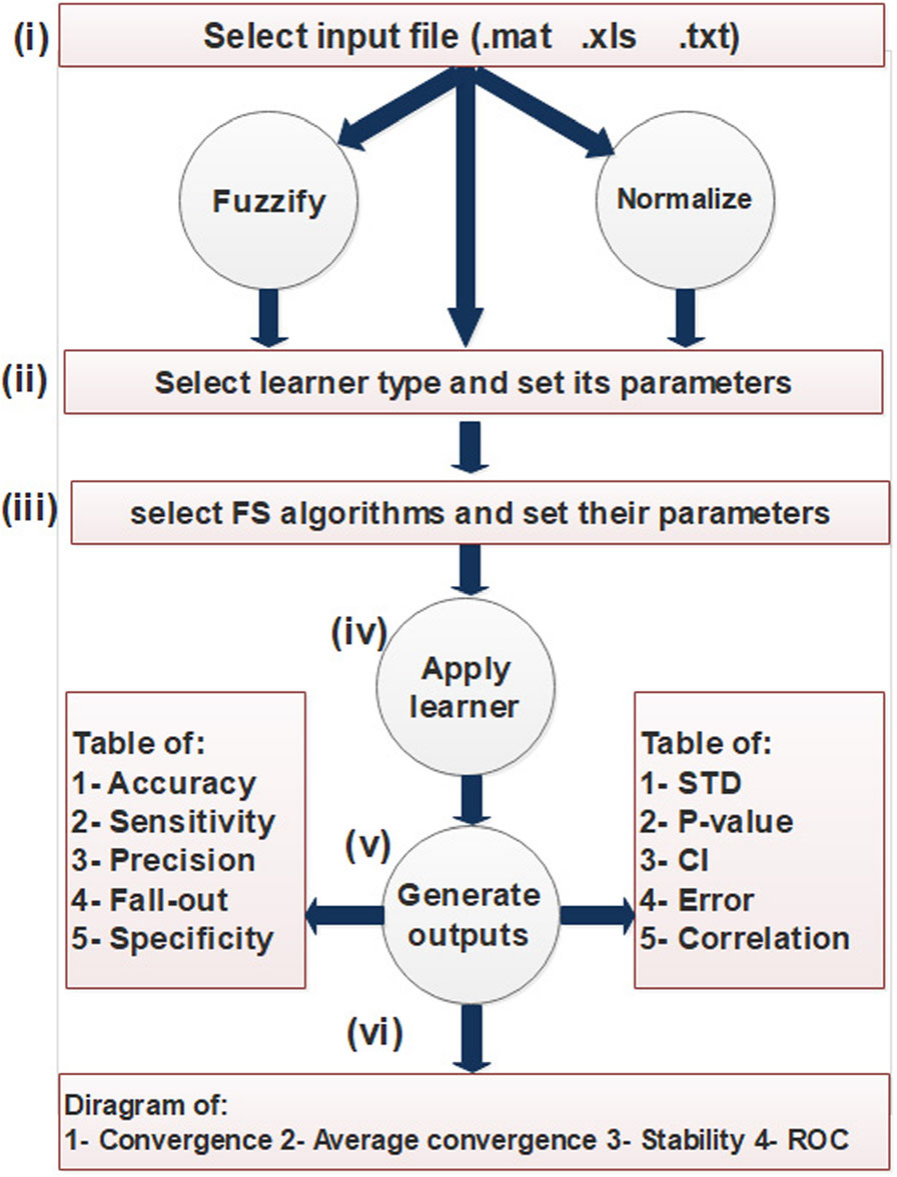
Framework of FeatureSelect

The framework of FeatureSelect is depicted in Fig. 2. The rectangles represent the interaction between FeatureSelect and the user, and the circles represent FeatureSelect processes.

FeatureSelect consists of six main parts: (i) an input file is selected, and is then fuzzified or normalised if necessary, since this can enhance the learner’s functionality; (ii) using a suitable GUI, one of the learners is chosen for classification or regression purpose, and its parameters is adjusted; (iii) one of the two available methods, filter or wrapper method, is selected for feature selection, and then the selected method parameters are determined. In wrapper methods, the list of optimisation algorithms is available. We investigated the performance of 33 optimisation algorithms and have selected 11 state-of-the-art algorithms based on their different natures and performance (Table 1).

**Table 1:** Implemented algorithms

Algorithm name	Abrr.	Operations on population	Pub.	Ref
World competitive contests	WCC	Attacking, shooting, passing, crossing	2016	[61]
League championship algorithm	LCA	Playing, transfer	2014	[62]
Genetic algorithm	GA	Crossover, mutation	1970	[63]
Particle swarm optimisation	PSO	Social behavior	1995	[64]
Ant colony optimisation	ACO	Edge selection, update pheromone	2006	[65]
Imperialist competitive algorithm	ICA	Revolution, absorb, move	2007	[66]
Learning automata	LA	Award, penalize	2003	[67]
Heat transfer optimisation	HTS	Molecules conductions	2015	[68]
Forest optimisation algorithm	FOA	Local seeding, global seeding	2014	[69]
Discrete symbiotic organisms search	DSOS	Mutualism, commensalism, parasitism	2017	[70]
Cuckoo optimisation algorithm	CUK	Eggs laying, eggs killing, eggs growing	2011	[71]

(iv) Selected features are evaluated by selected learner. For this purpose, three types of learner can be chosen and adjusted.

(v) *FeatureSelect* generates various types of results, based on the nature of the problem and selected method, and compares selected algorithms or methods with each other. The status of the executions and selected optimisation algorithms are available in the sixth section.

**Fig. 3:**
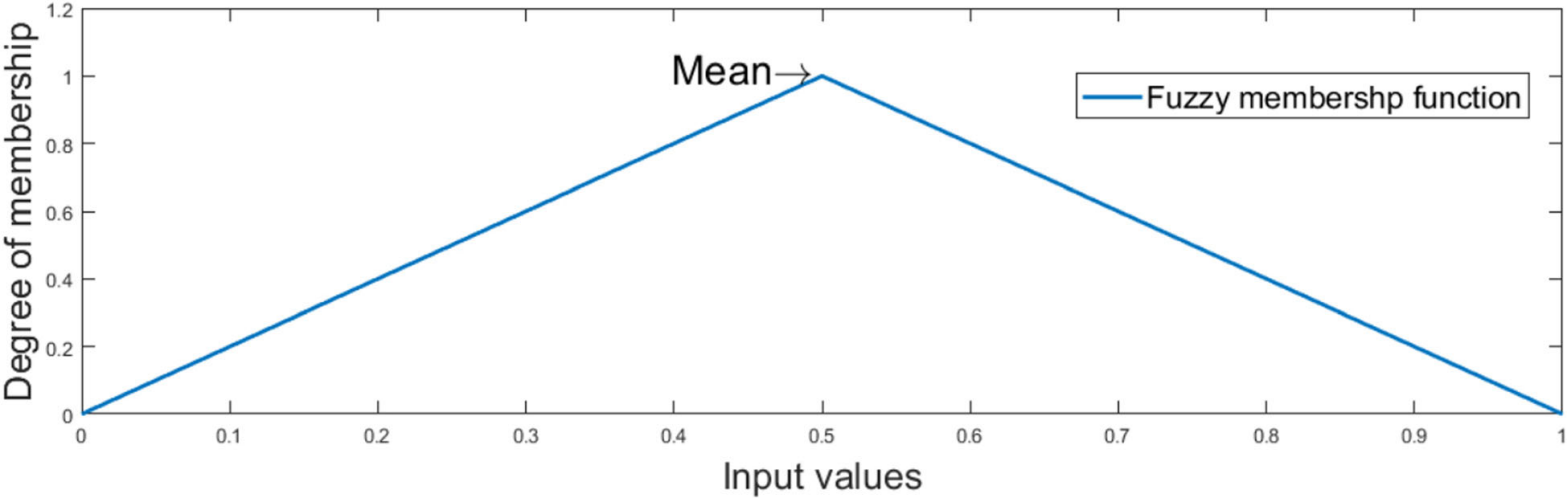
Fuzzy membership function

The relevant properties of FeatureSelect are described below:

- Data fuzzification and data normalisation capabilities are available. Data are converted to the range [0,1] in both the fuzzification and normalisation stages. TXT, XLS and MAT formats are acceptable as formats for the input file. Data normalisation is carried out as shown in Eq. 1.

1 $$ {\mathrm{v}}^{\hbox{'}}=\mathrm{low}+\frac{\left(v-v\mathit{\min}\right)\times \left( high- low\right)}{\left(v\max -v\min \right)} $$

where v’, v, vmax, vmin, high and low are the normalised value, the current value to be normalised, the maximum and minimum values of the group, and the higher and the lower bounds of the range, respectively. High and low are configured to one and zero respectively in FeatureSelect. Fuzzification is the process that convert scalar values to fuzzy values [50]. Figure 3 illustrates the fuzzy membership function used in FeatureSelect.

**Fig. 4:**
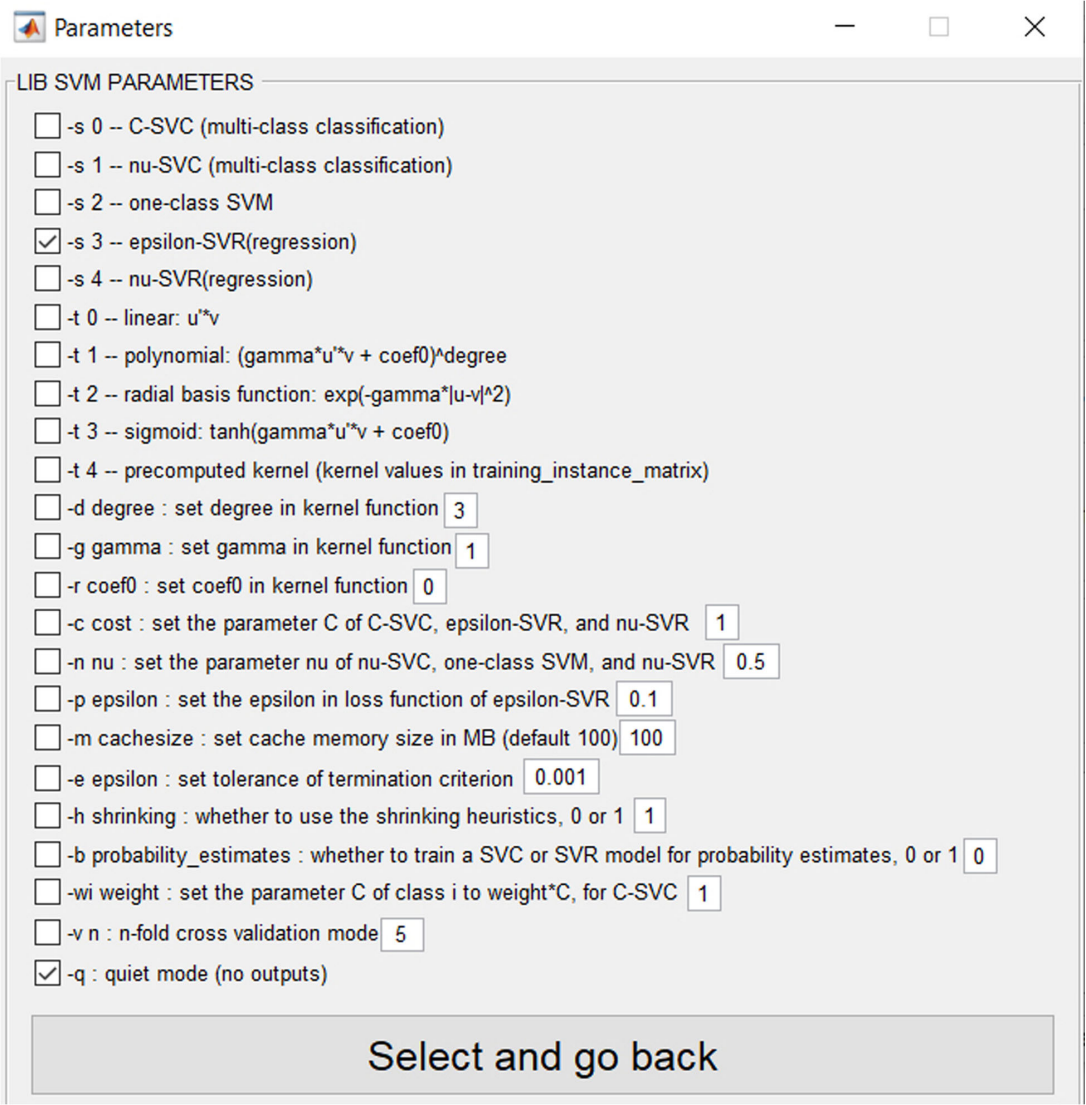
Parameters for LIBSVM in FeatureSelect

b) It provides a suitable graphical user interface for LIBSVM. For example, researchers can select LIBSVM’s learning parameters and apply them to their applications after selecting the input data (Fig. 4). If a researcher is unfamiliar with the training and testing functions in LIBSVM, he/she can easily use LIBSVM by clicking on the corresponding buttons.
c) Optimisation algorithms, which are used for feature selection, have been tested and the correctness of them has been examined. Researchers can select one or more of these optimisation algorithms using the relevant box.
d) A user can select different types of learners and feature selection methods, and employee them as ensemble feature selection method. For example, a user can reduce the number of available features by filter methods, and then can use optimisation algorithms or other methods in order to acquire better results.
e) After executing a selected algorithm in a regression problem, FeatureSelect automatically generates useful diagrams and tables, such as the error convergence, error average convergence, error stability, correlation convergence, correlation average convergence and correlation stability diagrams for the selected algorithms in. In classification problems, results include: the accuracy convergence, the accuracy average convergence, the accuracy stability, the error convergence, the error average convergence and the error stability. For both regression and classification problems, an XLS file is generated consisting of a number of selected features, including standard deviation, *P*-value, confidence interval (CI) and the significance of the generated results, and a TXT file containing detailed information such as the indices of the selected features. For classification problems, certain statistical results such as accuracy, precision, false positive rate, and sensitivity are generated. Eqs. 2 to 5 express how these measures are computed in FeatureSelect, where ACC, PRE, FPR and SEN are abbreviations for accuracy, precision, false positive rate and sensitivity, respectively.

2 $$ \mathrm{ACC}=\frac{\sum_{i=1}^n\left(\frac{TPi+ TNi}{TPi+ FNi+ FPi+ TNi}\right)\times Ci}{n} $$

3 $$ \mathrm{SEN}=\frac{\sum_{i=1}^n\left(\frac{TPi}{TPi+ FNi}\right)\times Ci}{n} $$

4 $$ \mathrm{PRE}=\frac{\sum_{i=1}^n\left(\frac{TPi}{TPi+ FPi}\right)\times Ci}{n} $$

5 $$ \mathrm{FPR}=\frac{\sum_{i=1}^n\left(\frac{FPi}{FPi+ TNi}\right)\times Ci}{n} $$

FeatureSelect obtains results for the average state since it can be applied to both binary and multiple classes of classification problems. In Eqs. 2 to 5, n, TP, TN, FP,,FN and C_i_ represent the number of classes, true positive, true negative, false positive, false negative and number of samples in ith class, respectively.

## Results

FeatureSelect has been developed in the MATLAB programming language (Additional file 1), since this is widely used in many research fields such as computer science, biology, medicine and electrical engineering. FeatureSelect can be installed and executed on several operating systems including Windows, Linux and Mac. Moreover, MATLAB-based softwares are open-source, allowing future researchers to add new features to the source code of FeatureSelect.

In this section, we will evaluate the performance of FeatureSelect, and compare its algorithms using various datasets. The eight datasets shown in Table 2 were employed to evaluate the algorithms used in FeatureSelect. Table 2 shows the reference, name, area, number of features (NOF), number of samples (NOS) and number of dataset classes (NOC). Four datasets correspond to classification problems, while the other datasets correspond to regression problems. Using the GitHub link (https://github.com/LBBSoft/FeatureSelect), these datasets can be downloaded.

**Table 2:** Datasets

Name	Type	Area	NOF	NOS	NOC	Ref
Social	Regression	Popularity prediction	59	200	–	[72]
DRUG	Regression	Drug design	221	56	–	[73]
AIR	Regression	Responses to gas multi sensors	15	9358	–	[74]
Energy	Regression	Energy use in low energy building	29	19,735	–	[75]
CARCINOM	Classification	Biology	9182	174	11	[76]
USPS	Classification	Hand written image data	256	9298	10	[76]
BASEHOCK	Classification	Text data	1993	4862	2	[76]
DRIVE	Classification	Driving in real scenario	606	6400	3	[77]

We ran FeatureSelect on a system with 12 GB of RAM, a COREi7 CPU and a 64-bit Windows 8.1 operating system. FeatureSelect automatically generates tables and diagrams for selected algorithms and methods. In this paper, we selected all algorithms and compared their operation. Each algorithm was run 30 individual times. Since optimisation algorithms operate randomly, it is advisable to evaluate them over at least 30 individual executions [51]. All the algorithms were run under the same conditions, for example calling an identical number of score functions. Accuracy and root mean squared error (RMSE) [52] were used as the score functions for classification and regression, respectively. The number of generations was set as 50 for all algorithms. We used WCC operators in LCA, since these improve the performance. The datasets (DS) and the name of the algorithm (AL) are shown in the first and second columns of Table 3 (classification datasets) and Table 4 (regression datasets). These tables, in which the best results of each column have been determined, represent certain statistical measures as ready reference for comparing the algorithms. These measures are as follows:

- NOF: Although the NOF was not applied to score functions, it can be restricted to an upper bound as a maximum number of features or genes in FeatureSelect. The maximum number of features was set as 400, 20, 10, 5, 5, 40, 10, and 5 for the CARCINOMA, BASEHOCK, USPS, DRIVE, AIR, DRUG, SOCIAL, and ENERGY datasets, respectively.
- Elapsed time (ET): After all algorithms were run 30 times, the best results were selected for each. The ET shows how much time in seconds elapsed in the execution for which the best result was obtained for an algorithm. Algorithms have different ETs due to their various stages.
- AC: This is a measure that states the rate of correctly predicted samples, relative to all the samples. The difference between AC and ACC is that ACC is an average accuracy for all classes, whereas AC is the accuracy of a specific class. The higher the accuracy, the better the answer.
- Accuracy standard deviation (AC_STD): This indicates how far the results differ from the mean of the results. It is therefore desirable that AC_STD is a minimum.
- CI: This represents a range of values, and the results are expected to fall into this range with a maximum specific probability. CI_L and CI_H stand for the lower and higher bounds on the confidence interval.
- *P*-value of accuracy (AC_P): The *p*-value is a statistical measurement that expresses the extent to which the obtained results are similar to random values. An algorithm with a minimum p-value is more reliable than others.
- Accuracy test statistic (AC_TS): TS is generally used to reject or accept a null hypothesis. When the TS is a maximum, the p-value is a minimum.
- Root mean squared error (ER or RMSE): ER is calculated using Eq. 6, where n, y_i_ and y’_i_ are the number of samples, and the predicted and label values, respectively. This measurement expresses the average difference between predicted and label values.

**Table 3:** Results obtained for classification datasets using SVM

DS	AL	NOF	ET	AC	AC_STD	AC_CI_L	AC_CI_H	AC_P	AC_TS	ER	ER_STD	ER_CI_L	ER_CI_H	ER_P	ER_TS
CARCINOM)40%, N)	WCC	319	108	27.35	0.28	27.15	27.37	4.33E-69	918.77	17.38	0.001	17.38	17.39	5.75E-94	18,272.5
	LCA	270	117	27.35	0.37	27.26	27.39	1.38E-65	869	17.38	0.002	17.38	17.39	1.96E-91	13,823.5
	GA	487	260	26.41	1.67	21.32	22.57	3.50E-34	71.6	17.42	0.06	17.57	17.62	6.57E-72	1435.54
	PSO	492	52	27.35	2.27	25.15	26.85	1.78E-32	62.47	17.38	0.09	17.4	17.47	6.12E-68	1047.51
	ACO	491	110	26.41	3.29	21.789	24.24	2.19E-26	38.29	17.42	0.13	17.51	17.6	2.13E-63	730.34
	ICA	488	79	27.35	1.11	25.21	26.04	2.55E-41	126.43	17.38	0.04	17.43	17.47	5.17E-77	2152.86
	LA	484	57	26.41	6.71	15.76	20.77	3.96E-15	14.9	17.42	0.26	17.65	17.85	1.47E-54	361.99
	HTS	480	43	26.41	3.68	18.97	21.72	1.69E-23	30.27	17.42	0.14	17.61	17.72	4.52E-62	657.31
	FOA	333	93	28.3	0.52	27.76	28.15	7.55E-52	291.89	17.42	0.07	17.36	17.41	1.11E-70	1301.99
	DSOS	363	78	27.35	0.23	26.38	26.56	4.79E-61	605.92	17.38	0.009	17.41	17.42	2.58E-96	9967.13
	CUK	408	111	27.35	0.53	26.78	27.17	3.06E-51	278.11	17.38	0.02	17.39	17.4	2.96E-86	4484.43
BASEHOCK(80%,O)	WCC	14	176	72	5.33	51.03	55.01	9.17E-31	54.48	0.18	0.05	0.45	0.49	2.93E-29	48.28
	LCA	15	140	75.25	6.57	53.91	58.82	6.49E-29	46.96	0.25	0.07	0.41	0.46	9.64E-26	36.35
	GA	20	327	48.75	0.87	46.18	46.82	6.60E-52	293.25	0.51	0.01	0.53	0.54	1.13E-53	337.4
	PSO	20	121	50.25	1.57	45.33	46.5	2.72E-44	160.12	0.5	0.02	0.53	0.55	2.37E-46	188.6
	ACO	20	140	47.75	1.1	45.01	45.83	1.09E-48	227.11	0.52	0.01	0.54	0.55	5.28E-51	272.95
	ICA	20	165	51	1.07	48.34	49.14	6.71E-50	250.04	0.49	0.01	0.51	0.52	1.55E-50	262.95
	LA	20	81	68.25	3.8	51.1	53.94	7.28E-35	75.61	0.32	0.04	0.46	0.49	1.33E-33	68.36
	HTS	20	65	47.5	0.89	45.32	45.98	2.25E-51	281.07	0.53	0.01	0.54	0.55	1.43E-53	334.63
	FOA	16	85	65.5	3.9	47	49.92	1.53E-33	68.02	0.35	0.04	0.5	0.53	2.59E-34	72.35
	DSOS	15	118	46	0.81	43.25	43.86	6.68E-52	293.13	0.54	0.01	0.56	0.57	3.65E-55	379.83
	CUK	18	138	66.25	3.04	51.37	53.64	1.16E-37	94.51	0.34	0.03	0.46	0.49	2.10E-36	85.48
USPS(80%, F)	WCC	10	13	85.15	0.19	84.93	85.39	4.60E-09	290.07	2.07	0.16	1.58	1.85	0.00001	28.5
	LCA	10	12	85.15	0.83	82.93	84.99	5.27E-09	226.64	2.15	0.26	2.06	2.7	0.00003	20.56
	GA	10	10	85.15	1.5	80.71	84.44	2.62E-08	122.97	2.56	0.38	2.1	3.05	0.00011	15.06
	PSO	10	6	87.13	2.05	82.01	87.1	8.33E-08	92.09	2.17	0.29	1.88	2.59	0.00006	17.34
	ACO	10	17	85.15	2.03	80.85	85.89	8.41E-08	91.87	2.91	0.48	1.57	2.77	0.00055	10.02
	ICA	10	7	86.14	2.05	80.02	85.12	9.16E-08	89.93	2.68	0.29	2.58	3.31	0.00002	22.37
	LA	10	16	89.11	2.89	83.54	90.71	2.88E-07	67.49	1.56	0.57	1.23	2.65	0.00161	7.59
	HTS	10	8	81.19	1.63	77.39	81.43	4.22E-08	109.14	3.43	0.62	3.2	4.74	0.00013	14.33
	FOA	10	9	83.17	1.29	80.38	83.58	1.47E-08	142	1.74	0.67	1.65	3.3	0.00113	8.33
	DSOS	10	14	82.18	2.85	74.28	81.36	4.32E-07	61.01	3.41	0.59	2.37	3.85	0.00003	11.69
	CUK	10	14	84.16	1.63	80.36	84.4	3.64E-08	113.22	2.1	0.68	1.46	3.16	0.001637	7.56
DRIVE)50%, N)	WCC	3	70	91.8	0.18	91.5	91.51	1.81E-76	2759	0.08	0.001	0.08	0.09	1.05E-45	185.46
	LCA	3	69	91.8	0.26	91.34	91.54	1.62E-75	1911.5	0.08	0.002	0.08	0.09	1.09E-45	178.97
	GA	3	16	91.8	0.33	90.95	91.2	1.67E-72	1505.2	0.08	0.002	0.09	0.09	2.93E-43	147.51
	PSO	3	6	91.26	0.88	88.63	89.29	6.05E-60	555.22	0.09	0.01	0.11	0.11	1.06E-33	68.89
	ACO	3	34	91.26	0.93	88.65	89.34	2.93E-59	525.82	0.09	0.01	0.11	0.11	5.69E-33	65
	ICA	3	9	91.8	0.74	90.72	91.28	2.41E-62	671.77	0.08	0.01	0.09	0.09	3.05E-33	66.42
	LA	3	18	91.26	1.26	89.04	89.98	1.92E-55	388.32	0.09	0.01	0.1	0.11	1.58E-28	45.52
	HTS	3	26	90.71	0.65	88.55	89.04	1.24E-63	744.03	0.09	0.01	0.11	0.11	1.41E-37	93.86
	FOA	2	41	91.26	0.78	88.54	89.13	2.21E-61	622.33	0.09	0.01	0.11	0.11	2.73E-35	78.22
	DSOS	3	52	91.26	0.53	88.45	88.85	3.12E-66	914.72	0.09	0.01	0.11	0.12	2.35E-40	117.09
	CUK	3	67	91.8	1.3	89.33	90.3	3.66E-55	379.77	0.08	0.01	0.1	0.11	7.78E-28	43.05

**Table 4:** Results obtained for regression datasets using SVM

DS	AL	NOF	ET	ER	ER_STD	ER_CI_1	ER_CI_2	ER_P	ER_TS	CR	CR_STD	CR_CI_1	CR_CI_2	CR_P	CR_TS
AIR(80%,O)	WCC	5	105	0.02	0.00	0.02	0.02	0	5.3E+ 15	0.60	0.00	0.60	0.60	0	1.0E+ 15
	LCA	5	164	0.02	0.00	0.02	0.02	1.0E-70	1306	0.60	0.00	0.60	0.60	1.25E-76	2088.68
	GA	5	73	0.02	0.00	0.02	0.02	1.3E-70	1295.2	0.60	0.01	0.59	0.60	1.08E-54	365.92
	PSO	5	39	0.02	0.00	0.02	0.02	1.9E-55	387.94	0.60	0.02	0.58	0.60	2.18E-42	137.64
	ACO	5	167	0.02	0.00	0.02	0.02	8.7E-54	340.36	0.60	0.04	0.57	0.60	2.68E-35	78.28
	ICA	5	41	0.02	0.00	0.02	0.02	6.7E-61	598.97	0.60	0.00	0.60	0.60	2.37E-69	1171.79
	LA	5	64	0.02	0.00	0.02	0.02	7.5E-60	551.02	0.60	0.04	0.57	0.60	2.27E-34	72.69
	HTS	4	64	0.02	0.00	0.02	0.02	3.7E-59	521.16	0.60	0.03	0.60	0.63	2.9E-39	107.35
	FOA	5	332	0.02	0.00	0.02	0.02	4.3E-62	658.04	0.60	0.02	0.59	0.60	4.85E-46	184.01
	DSOS	5	139	0.02	0.00	0.02	0.02	7.1E-53	316.65	0.60	0.03	0.55	0.58	1.14E-37	94.57
	CUK	5	173	0.02	0.00	0.02	0.02	2.1E-68	1086	0.60	0.00	0.60	0.60	2.6E-74	1737.29
DRUG(80%,N)	WCC	32	140	0.01	0.00	0.01	0.01	2.7E-26	38.01	0.97	0.01	0.96	0.96	1.61E-65	864.45
	LCA	23	115	0.00	0.00	0.01	0.01	3.3E-25	34.80	0.97	0.00	0.96	0.97	4.33E-72	1456.43
	GA	38	48	0.01	0.00	0.02	0.02	1.0E-31	58.83	0.95	0.01	0.94	0.95	1.67E-56	422.49
	PSO	36	47	0.01	0.00	0.01	0.01	9.3E-24	30.92	0.96	0.01	0.96	0.96	3.15E-63	720.56
	ACO	36	141	0.01	0.00	0.02	0.02	9.4E-24	30.91	0.97	0.01	0.95	0.96	1.16E-55	395.13
	ICA	35	38	0.01	0.00	0.02	0.02	6.7E-30	50.81	0.96	0.01	0.95	0.96	5.35E-61	603.64
	LA	30	95	0.00	0.00	0.00	0.00	4.1E-24	31.84	0.98	0.00	0.97	0.97	3.35E-71	1357.20
	HTS	32	98	0.01	0.00	0.02	0.03	3.8E-25	34.63	0.95	0.01	0.94	0.95	4.88E-57	440.77
	FOA	20	99	0.00	0.00	0.01	0.01	1.9E-18	19.88	0.97	0.01	0.96	0.96	6.19E-66	893.35
	DSOS	18	119	0.01	0.00	0.02	0.02	7.1E-29	46.80	0.96	0.01	0.95	0.96	3.24E-63	719.88
	CUK	24	152	0.01	0.00	0.01	0.01	1.8E-30	53.15	0.97	0.01	0.96	0.97	4.68E-65	833.19
SOCIAL (80%,F)	WCC	8	121	0.02	0.00	0.01	0.02	3.44E-08	229.53	0.51	0.07	0.30	0.64	0.006725	12.13
	LCA	8	135	0.02	0.00	0.01	0.02	4.66E-05	146.54	0.54	0.02	0.48	0.56	0.00033	55.01
	GA	10	68	0.02	0.00	0.02	0.02	0.000558	42.33	0.36	0.04	0.23	0.44	0.005372	13.59
	PSO	10	91	0.02	0.00	0.02	0.02	8.69E-05	107.26	0.39	0.05	0.24	0.47	0.00549	13.44
	ACO	10	153	0.02	0.00	0.02	0.02	0.000394	50.35	0.31	0.05	0.17	0.42	0.010204	9.82
	ICA	9	76	0.02	0.00	0.02	0.02	0.00017	76.61	0.37	0.01	0.36	0.39	6.79E-05	121.39
	LA	10	93	0.02	0.00	0.01	0.02	0.000485	45.39	0.53	0.02	0.45	0.57	0.000754	36.40
	HTS	8	93	0.02	0.00	0.02	0.02	6.75E-05	121.73	0.36	0.03	0.23	0.41	0.003921	15.92
	FOA	8	86	0.02	0.00	0.01	0.03	0.010557	9.66	0.45	0.16	0.10	0.70	0.083971	3.23
	DSOS	8	122	0.02	0.00	0.02	0.03	0.001028	31.17	0.25	0.04	0.11	0.31	0.012132	9.00
	CUK	8	93	0.02	0.00	0.02	0.02	0.000439	47.70	0.35	0.03	0.26	0.39	0.002276	20.93
ENERGY(60%,O)	WCC	5	64	0.08	0.00	0.08	0.08	6.03E-80	2717.4	0.5	0	0.4	0.4	1.19E-35	80.49
	LCA	5	82	0.08	0.00	0.08	0.08	1.60E-83	3609.2	0.5	0	0.4	0.4	6.82E-33	64.59
	GA	5	23	0.08	0.00	0.08	0.08	2.70E-75	1878.2	0.4	0	0.3	0.4	3.46E-29	48
	PSO	5	25	0.08	0.00	0.08	0.08	7.82E-70	1217.4	0.3	0.1	0.3	0.3	3.16E-23	29.61
	ACO	5	52	0.08	0.00	0.08	0.08	1.34E-63	742.04	0.4	0.1	0.2	0.3	1.54E-17	18.4
	ICA	5	57	0.08	0.00	0.08	0.08	4.89E-79	2528.3	0.5	0	0.4	0.4	1.55E-31	57.95
	LA	5	24	0.08	0.00	0.08	0.08	1.57E-73	1632.7	0.5	0	0.4	0.4	1.07E-29	49.99
	HTS	4	27	0.08	0.00	0.08	0.08	1.08E-66	948.73	0.4	0.1	0.3	0.3	1.78E-18	19.94
	FOA	5	30	0.08	0.00	0.08	0.08	2.20E-66	925.79	0.5	0.1	0.3	0.3	1.97E-20	23.51
	DSOS	5	42	0.08	0.00	0.08	0.08	3.70E-66	909.35	0.4	0.1	0.3	0.3	6.59E-24	31.31
	CUK	5	80	0.08	0.00	0.08	0.08	2.33E-80	2807.9	0.5	0	0.4	0.4	6.99E-32	59.58

6 $$ \mathrm{ER}=\sqrt{\frac{\left( yi-{y}^{\hbox{'}}i\right)}{n}} $$

i) Error standard deviation (ER_STD): In the same way as AC_STD, ER_STD indicates how far the RMSE differs from the average RMSE when 30 individual executions are performed. The lower the ER_STD, the closer the obtained results.
j) Squared correlation coefficient (CR): The correlation (R) determines the connectivity between the predicted values and label values. CR is calculated based on R^2^. We expect the CR to increase when the error decreases.

The concepts between (ER_CI_L and CR_CI_L and AC_CI_L), between (ER_CI_H and CR_CI_H and AC_CI_H), between (ER_STD and CR_STD and AC_STD), between (AC_P and ER_P and CR_P), and finally between (AC_TS and ER_TS and CR_TS) are alike. In addition to the name of the dataset, the training data percentage and an input data type are specified. Three input data types were used: fuzzified (F), normalised, (N) and ordinary (O).

FeatureSelect generates diagrams for the ACC, average of the ACC and the stability of the ACC for classification datasets. In addition, it generates diagrams of the ER, average ER and stability of the ER for both classification and regression datasets.

**Fig. 5:**
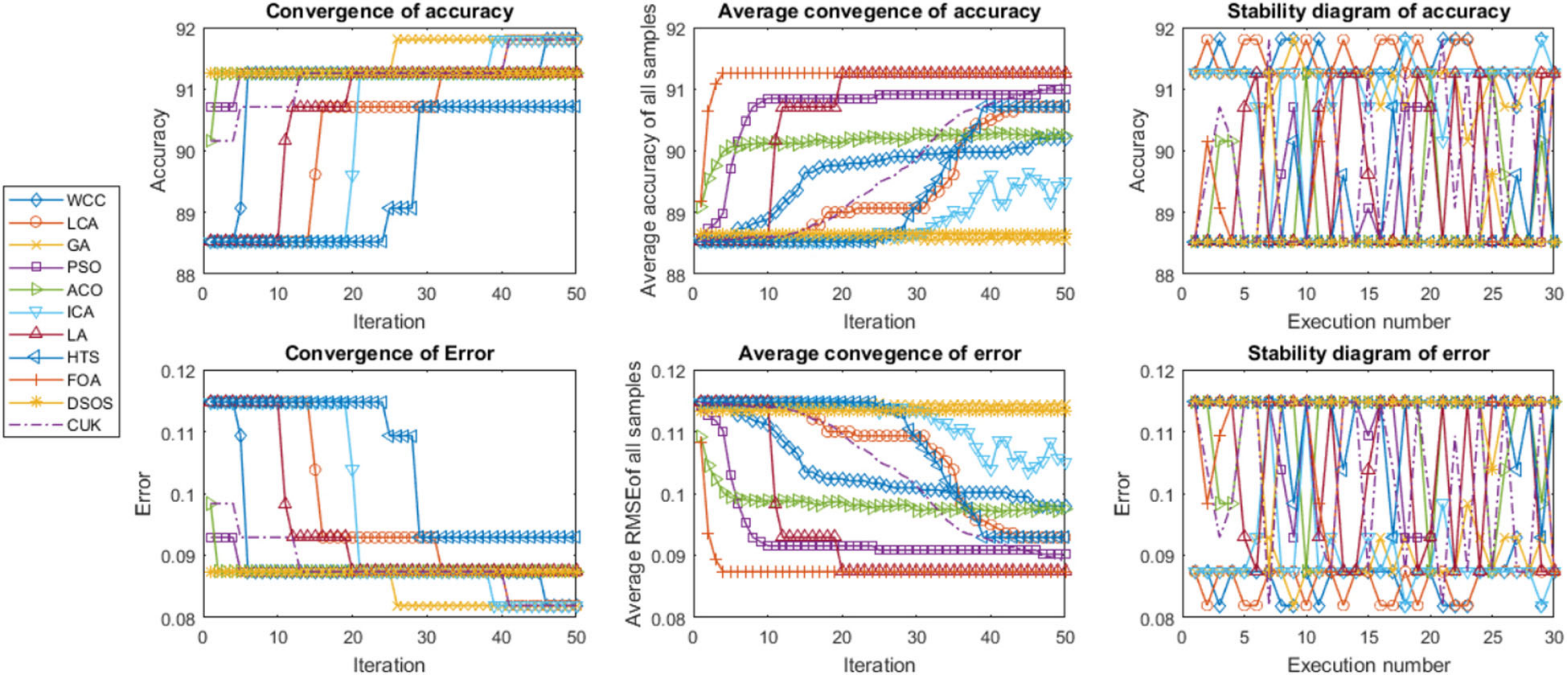
Diagrams generated for the DRIVE dataset using SVM. These diagrams compare the algorithms performances against each other based on accuracy and error scores. For every score, convergence, average convergence, and stability diagrams have been shown. Given the results on the DRIVE dataset, the performances of WCC, GA, LCA, and LA are better than the others

**Fig. 6:**
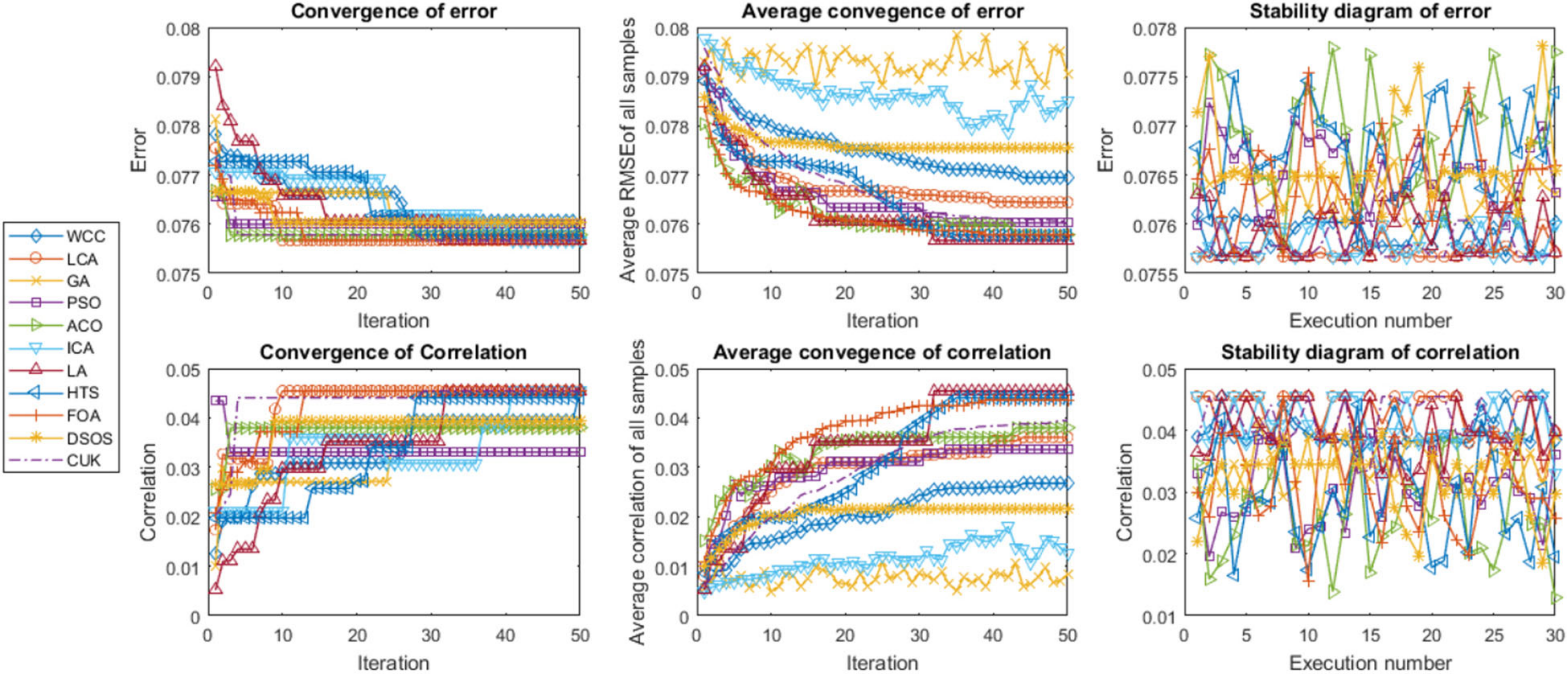
Diagrams generated for the ENERGY dataset using SVR. These diagrams compare the algorithms performances against each other based on RMSE and correlation scores. For every score, convergence, average convergence, and stability diagrams have been shown. Given the results on the ENERGY dataset, the performances of CUK, HTS, LCA, and LA are proper than the others

**Fig. 7:**
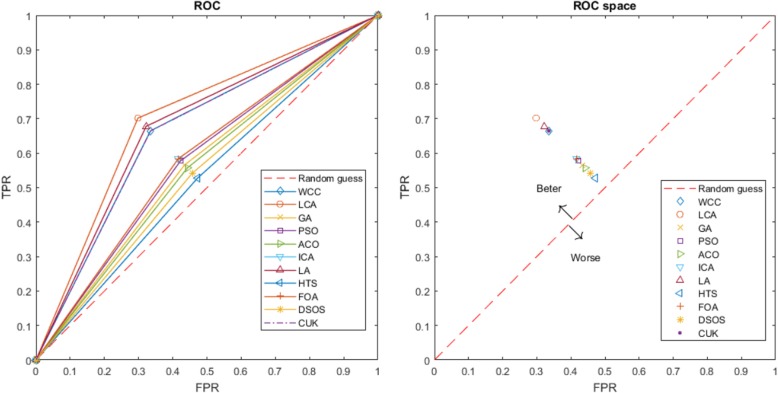
ROC curve and ROC space for the algorithms used based on SVM

The criteria used to evaluate the optimisation algorithms were convergence, average convergence and stability. These measures indicate whether or not the algorithms have been correctly implemented. Figures 5 and 6 illustrate instances of FeatureSelect outputs based on the mentioned criteria. The convergence mean is that the answers must be improved when the number of iterations or time dedicated to the algorithms is increased. For example, we observe that the ER decreases and the CR and ACC increase with a higher number of iterations. From convergence point of view, all of the algorithms increase the accuracy and correlation, and reduce the error. Although all of them have generated acceptable results, LA, LCA, WCC and GA are suitable than others. In addition to convergence, there is the concept of average convergence. The difference between the two is that the convergence is obtained by extracting the best answer at the end of each iteration, whereas average convergence is calculated based on the mean of potential solution scores at the end of each iteration. As it is observable, all of the potential answers generated by algorithms except GA and ICA are improving when the iteration is increased. In order to improve the performance of GA, we replace some of the worst results with randomly created answers at the end of each iteration. Also, absorb operator of ICA makes some countries worse or better than their previous status. Hence, the average convergence of GA and ICA may not have ascending or descending form. Stability diagrams indicate how the results fluctuate from a forward line in the individual executions. An algorithm can be said to be better than others if its results lie on the forward line and if the mean of its results is better than those of other algorithms. The results shown in Tables 3 and 4 have been calculated based on the stability results. FeatureSelect also generates several addition outputs for classification datasets, as follows:

- Essential statistical measurements: These measures are shown in Eqs. 2 to 5. Table 5 presents these statistical measures for all datasets.
- Receiver operating characteristic (ROC) curve: This is usually used for binary classification, but has been extended here to multi-class classification. The ROC is a graphical plot that indicates the diagnostic ability of a classifier. The horizontal axis is FPR (1-specificity) and the vertical axis is TPR (true positive rate or sensitivity) [53]. The ROC curve and ROC space for the algorithms for the USPS dataset are shown in Fig. 7 as an example of FeatureSelect’s output for classification datasets.

**Table 5:** Essential statistical measurements for all classification datasets

DS	AL_NAME	SEN	PRE	FPR	ACC	DS	AL_NAME	SEN	PRE	FPR	ACC
CARCINOM(80%,N)	WCC	0.68	0.60	0.02	0.76	USPS(80%,O)	WCC	0.82	0.86	0.02	0.85
	LCA	0.68	0.60	0.02	0.76		LCA	0.82	0.83	0.02	0.85
	GA	0.68	0.60	0.02	0.75		GA	0.83	0.86	0.02	0.85
	PSO	0.68	0.60	0.02	0.76		PSO	0.87	0.88	0.02	0.87
	ACO	0.68	0.60	0.02	0.75		ACO	0.85	0.85	0.02	0.85
	ICA	0.68	0.60	0.02	0.76		ICA	0.81	0.89	0.02	0.86
	LA	0.68	0.60	0.02	0.75		LA	0.89	0.89	0.01	0.89
	HTS	0.68	0.60	0.02	0.58		HTS	0.79	0.82	0.03	0.81
	FOA	0.68	0.60	0.02	0.77		FOA	0.81	0.84	0.02	0.83
	DSOS	0.68	0.60	0.02	0.76		DSOS	0.80	0.80	0.02	0.82
	CUK	0.68	0.60	0.02	0.76		CUK	0.82	0.84	0.02	0.84
BASEHOCK(80%,F)	WCC	0.66	0.89	0.33	0.72	DRIVE(80%,N)	WCC	0.56	0.81	0.24	0.92
	LCA	0.70	0.83	0.30	0.75		LCA	0.56	0.81	0.24	0.92
	GA	0.57	0.72	0.43	0.49		GA	0.56	0.81	0.24	0.92
	PSO	0.58	0.71	0.42	0.50		PSO	0.52	0.80	0.25	0.91
	ACO	0.56	0.72	0.44	0.48		ACO	0.52	0.80	0.25	0.91
	ICA	0.58	0.72	0.42	0.51		ICA	0.56	0.81	0.24	0.92
	LA	0.68	0.67	0.32	0.68		LA	0.52	0.80	0.25	0.91
	HTS	0.53	0.71	0.47	0.44		HTS	0.33	0.63	0.33	0.89
	FOA	0.58	0.75	0.42	0.66		FOA	0.52	0.80	0.25	0.91
	DSOS	0.54	0.72	0.46	0.46		DSOS	0.52	0.80	0.25	0.91
	CUK	0.66	0.66	0.34	0.66		CUK	0.56	0.81	0.24	0.92

Like the ROC curve, the ROC space represents the trade-offs between TPR and FPR. A point that is closer to the left and the top represents an algorithm with better diagnostic ability; for example, LCA has the best diagnostic ability for the USPS dataset.

In overall evaluation, we compare the performance of the FeatureSelect algorithms. The values in Tables 6, 7 and 8 are a summary of those in Tables 3, 4 and 5 respectively (the average for table), and allow an overall comparison of the algorithms used in FeatureSelect. LCA has selected 74.5 features in the average state on four classification datasets. Although the time orders are the same for all algorithms, the average elapsed time for four classification datasets is 35.5 for HTS. LCA and WCC show similar operation, but the accuracy of LCA is better than that of WCC. Its accuracy confidence interval is also more acceptable than that of the others. We show the AC_P and ER_P using three floating digits.

**Table 6:** Summary of results for all classification datasets

AL	NOF	ET	AC	AC_STD	AC_CI_L	AC_CI_H	AC_P	AC_TS	ER	ER_STD	ER_CI_L	ER_CI_H	ER_P	ER_TS
WCC	86.50	91.75	69.08	1.50	63.65	64.82	0.000	1005.58	4.93	0.05	4.94	4.96	0.000	4633.69
LCA	74.50	84.50	69.89	2.01	63.86	65.69	0.000	763.53	4.97	0.08	4.98	5.16	0.000	3514.85
GA	130.00	153.25	63.03	1.09	59.79	61.26	0.000	498.26	5.14	0.11	5.07	5.33	0.000	483.88
PSO	131.25	46.25	64.00	1.69	60.28	62.44	0.000	217.48	5.04	0.10	4.98	5.18	0.000	330.59
ACO	131.00	75.25	62.64	1.84	59.07	61.33	0.000	220.77	5.24	0.16	4.93	5.26	0.000	269.58
ICA	130.25	65.00	64.07	1.24	61.07	62.90	0.000	284.54	5.16	0.09	5.15	5.35	0.000	626.15
LA	129.25	43.00	68.76	3.67	59.86	63.85	0.000	136.58	4.85	0.22	4.86	5.28	0.000	120.87
HTS	128.25	35.50	61.45	1.71	57.56	59.54	0.000	291.13	5.37	0.20	5.37	5.78	0.000	275.03
FOA	90.25	57.00	67.06	1.62	60.92	62.70	0.000	281.06	4.90	0.20	4.91	5.34	0.000	365.22
DSOS	97.75	65.50	61.70	1.11	58.09	60.16	0.000	468.70	5.36	0.15	5.11	5.49	0.000	2618.94
CUK	109.75	82.50	67.39	1.63	61.96	63.88	0.000	216.40	4.98	0.19	4.85	5.29	0.000	1155.13

**Table 7:** Summary of results for all regression datasets

AL	NOF	ET	ER	ER_STD	ER_CI_1	ER_CI_2	ER_P	ER_TS	CR	CR_STD	CR_CI_1	CR_CI_2	CR_P	CR_TS
WCC	12.5	107.5	0.033	0.000	0.030	0.033	0.000	1.3E+ 15	0.65	0.020	0.615	0.640	0.000	2.5E+ 14
LCA	10.25	124	0.030	0.000	0.030	0.033	0.000	1274.13	0.65	0.005	0.610	0.633	0.000	916.1775
GA	14.5	53	0.033	0.000	0.035	0.035	0.000	818.640	0.57	0.015	0.515	0.598	0.001	212.5
PSO	14.00	50.5	0.033	0.000	0.033	0.033	0.000	435.880	0.56	0.045	0.520	0.583	0.001	225.3125
ACO	14.00	128.25	0.033	0.000	0.035	0.035	0.000	290.915	0.57	0.050	0.473	0.570	0.003	125.4075
ICA	13.50	53	0.033	0.000	0.035	0.035	0.000	813.673	0.60	0.005	0.578	0.588	0.000	488.6925
LA	12.50	69	0.030	0.000	0.028	0.030	0.000	565.238	0.65	0.015	0.598	0.635	0.000	379.07
HTS	12.00	70.5	0.033	0.000	0.035	0.038	0.000	406.563	0.57	0.043	0.518	0.573	0.001	145.995
FOA	9.50	136.75	0.030	0.000	0.030	0.035	0.003	403.343	0.63	0.073	0.488	0.640	0.021	276.025
DSOS	9.00	105.5	0.033	0.000	0.035	0.038	0.000	325.99	0.55	0.045	0.478	0.538	0.003	213.69
CUK	10.50	124.5	0.033	0.000	0.033	0.033	0.000	998.68	0.60	0.010	0.555	0.590	0.001	662.7475

**Table 8:** Summary of essential statistical criteria for all classification datasets

AL_NAME	SEN	PRE	FPR	ACC
WCC	0.6800	0.7900	0.1525	0.8125
LCA	0.6900	0.7675	0.1450	0.8200
GA	0.6600	0.7475	0.1775	0.7525
PSO	0.6625	0.7475	0.1775	0.7600
ACO	0.6525	0.7425	0.1825	0.7475
ICA	0.6575	0.7550	0.1750	0.7625
LA	0.6925	0.7400	0.1500	0.8075
HTS	0.5825	0.6900	0.2125	0.6800
FOA	0.6475	0.7475	0.1775	0.7925
DSOS	0.6350	0.7300	0.1875	0.7375
CUK	0.6800	0.7275	0.1550	0.7950

These values are identical for all algorithms, indicating that the performance of the algorithms is not random. For all classification datasets, FOA reaches a minimum value of ER. Therefore, it is proper than other algorithms in ER point of view. We also observe that WCC operates better than the other algorithms in terms of ER_TS, CR, CR_CI, CR_P and CR_TS.

The DSOS algorithm selects nine features in the average state for all regression datasets. The elapsed time for PSO in which the best answer has been obtained was lowest for this algorithm. LCA, LA and FOA are algorithms which their functional are the same and proper than other algorithms. It is also obvious that LA has the best confidence interval of all alternative approaches. Except for FOA, which has an ER_P value of 0.003, ER_P is identical for all algorithms to three decimal places. In the same way as CR_CI, CR_P and CR_TS for all regression datasets, the highest ER_TS value was achieved by WCC. WCC, LCA and LA achieved the maximum value of correlation (CR) for all regression datasets.

SEN, PRE, FPR, and ACC are the most important comparison criteria for classification problems. A summary of Table 5 is shown in Table 8, which indicates that LCA obtains the best results in terms of FPR and ACC, and LA achieves the best result for SEN. WCC also acquires the best result for PRE on average.

In a comprehensive comparison, we evaluate the performance of all algorithms and methods on BSEHOCK dataset that is larger than others. Unlike previous experiments which are based on single objective (ACC) score; this one is based on multi objective score for wrapper methods. In Table 9 in which the best values of each column have been determined; the results are observable for SVM, ANN and DT learner. PCRR, LAP, ENT and MI are abbreviation for pearson correlation, laplacian, entropy and mutual information respectively in Table 9. As it is observed, every classifier and every feature selection method have their own attitude toward the data. Therefore, a user can apply various methods and algorithms along with different learners, and then can select the features which satisfy his/hers requirements. Also, it is possible that a user employee ensemble.

**Table 9:** A comprehensive comparison of all methods

AL	Learner = SVM					Learner = ANN					Learner = Decision tree				
	SEN	SPC	PRE	FPR	ACC	SEN	SPC	PRE	FPR	ACC	SEN	SPC	PRE	FPR	ACC
WCC	0/92	0/25	0/43	0/75	0/51	0/94	0/21	0/63	0/79	0/63	0/45	**0/69**	0/34	**0/31**	**0/52**
LCA	0/92	0/25	0/43	0/75	0/51	0/85	0/24	0/70	0/76	0/70	0/46	0/67	**0/36**	0/33	0/50
GA	0/92	0/25	0/43	0/75	0/51	0/96	0/02	0/63	0/98	0/63	0/44	0/61	0/33	0/39	0/45
PSO	0/92	0/25	0/43	0/75	0/51	**1/00**	0/00	0/65	1/00	0/65	0/44	0/63	0/31	0/37	0/47
ACO	0/92	0/25	0/43	0/75	0/51	0/97	0/14	0/72	0/86	0/72	0/43	0/60	0/31	0/40	0/43
ICA	0/92	0/25	0/43	0/75	0/51	**1/00**	0/00	0/70	1/00	0/70	0/44	0/62	0/33	0/38	0/45
LA	0/92	0/25	0/43	0/75	0/51	**1/00**	0/00	**0/73**	1/00	**0/73**	0/45	0/63	**0/36**	0/37	0/42
HTS	0/93	0/21	0/42	0/79	0/49	0/90	0/33	0/55	0/67	0/55	0/43	0/57	0/31	0/43	0/41
FOA	0/90	**0/32**	**0/46**	**0/68**	**0/54**	0/94	0/22	0/67	0/78	0/67	0/44	0/63	0/34	0/37	0/46
DSOS	0/92	0/25	0/43	0/75	0/51	0/74	**0/51**	0/67	**0/49**	0/67	0/44	0/61	0/34	0/39	0/44
CUK	0/92	0/25	0/43	0/75	0/51	0/83	0/40	0/65	0/60	0/65	0/43	0/59	0/28	0/41	0/43
PCRR	**0/98**	0/04	0/36	0/96	0/43	0/96	0/02	0/67	0/98	0/67	0/43	0/28	0/15	0/72	0/17
LAP	0/94	0/17	0/40	0/83	0/48	0/77	0/35	0/67	0/65	0/67	0/44	0/39	0/18	0/61	0/27
ENT	0/94	0/17	0/40	0/83	0/48	**1/00**	0/00	0/67	1	0/67	0/43	0/61	0/30	0/39	0/45
MI	1/00	0/00	0/35	1/00	0/41	**1/00**	0/00	0/68	1	0/68	**0/50**	0/00	0/00	1/00	0/00
Fisher	1/00	0/00	0/35	1/00	0/41	0/98	0/06	0/67	0/94	0/67	**0/50**	0/00	0/00	1/00	0/00

Boldface values indicate the best-obtained results of each criterion for every learner

## Discussion

Feature selection is one the most important steps in machine learning applications. For this purpose, many tools and methods have been introduced by researchers. For example, a feature weighting tool for unsupervised applications [54] and Weka machine learning tool [55] have been developed. However, the main limitation of these tools like mRMR [56] and mRMD [57] is that they are based on filter methods which only consider the relation among features and disregard interaction between feature selection algorithm and learner. As another example, we can mention a wrapper feature selection tool which is based on genetic algorithm [58]. Although time complexity of wrapper methods are higher than filter ones, these methods can lead better results; and it is valuable to spend more time. In this paper, we proposed a machine learning software named FeatureSelect that includes three types of popular learners (SVM, ANN and DT). In addition, two types of feature selection method are available in it. First method is wrapper method that is based on optimisation algorithms. Eleven state-of-art optimisation algorithms have been selected based on their popularity, novelty and functionality, and then implemented in FeatureSelect. Second type is the filter method which is based on Pearson correlation, entropy, Laplacian, mutual information and fisher scores. A user can also combine existing methods and algorithms, and then use them as ensemble or hybrid method like hybrid feature selection methods [59]. For example, a user can confine a number of features to specific threshold using filter methods. After it, the user can use wrapper methods along with an agile learner such as SVM or DT for acquiring an optimal subset of features, and finally engage and test ANN with enhancing a number of training iterations to obtain suitable model. There are also some other application-specific tools like iFeature [60] which is used for extracting and selecting features from protein and peptide sequences. Although iFeature includes a web server besides a stand-alone tool, FeatureSelect is the general software and provides different capabilities like hybrid feature selection and ensemble learning based on various states of combining filter and wrapper methods. In order to show capabilities of FeatureSelect, we applied it on various datasets with different sizes in multiple areas. The results show that every algorithm and every learner has its attitude relative to data, and algorithms’ performances vary on different data. In another comprehensive experiment, we applied all of algorithms and learners of FeatureSelect on the BASEHOCK dataset with multi-objective score function. Although filter methods are quicker than wrapper methods, the acquired results present that wrapper methods’ performance are proper than the filter methods.

## Conclusions

In this paper, a new software application for feature selection is proposed. This software is called FeatureSelect, and can be used in fields such as biology, image processing, drug design and numerous other domains. FeatureSelect selects a subset of features using optimisation algorithms with considering different score functions and then transmits these to the learner. SVM, ANN and DT are used here as a learner that can be applied to classification and regression datasets. Since LIBSVM is a library for SVM and provides a wide range of options for classification and regression problems, we developed FeatureSelect based on this library. Researchers can apply FeatureSelect to any dataset using three types of learners and two types of feature selection methods and obtain various tables and diagrams based on the nature of the dataset. It is also possible to combine the methods and algorithms as ensemble method. FeatureSelect was applied to eight datasets with differing scope and size. We then compared the performance of the algorithms in FeatureSelect to these datasets and presented some examples of the outputs in the form of tables and diagrams. Although the algorithms and feature selection methods have different functionality for different datasets, WCC, LCA, LA and FOA are the algorithms having proper functionality than others, and wrapper methods lead better results than filter methods.

## Additional file

Additional file 1: The supplementary file. It consists of source codes. FeatureSelect has been implemented in MATLAB and is free open source software. Therefore, users can change or improve it. The modified versions of it will be uploaded to the GItHub repository. Also, three types of stand-alone versions of FeatureSelect, including WIN 64-bit, java, and python packages, are available. (ZIP 151 mb)

## Abbreviations

ACC: Accuracy
ACO: Ant Colony Optimization
ANN: Artificial Neural Network
CUK: Cuckoo algorithm
DSOS: Discrete Symbiotic Optimization Search
ER: Error
FOA: Forest Optimization Algorithm
FPR: False Positive Rate
FS: Feature Selection
GA: Genetic Algorithm
HTS: Heat Transfer Optimization
ICA: Imperialist Competitive Algorithm
LA: Learning Automata
LCA: League Championship Algorithm
PRE: Precision
PSO: Particle Swarm Optimization
SEN: Sensitivity
SPC: Specificity
SVM: Support Vector Machine
WCC: World Competitive Contest Algorithm
