# Supplementary material for: FeatureSelect: a software for feature selection based on machine learning approaches
Source: BMC Bioinformatics. 2019 Apr 3;20:170. doi: 10.1186/s12859-019-2754-0 (PMC6446290; doi:10.1186/s12859-019-2754-0)
Supplement: Supplementary file 1 — The supplementary file. It consists of source codes. FeatureSelect has been implemented in MATLAB and is free open source software. Therefore, users can change or improve it. The modified versions of it will be uploaded to the GItHub repository. Also, three types of stand-alone versions of FeatureSelect, including WIN 64-bit, java, and python packages, are available. (ZIP 151 mb) [file 12859_2019_2754_MOESM1_ESM.zip › Additional file/MATLAB_version.zip!/FeatureSelect/matlab/help/Tutorial for FEATURESELECT.pdf]

Tutorial for *FEATURESELECT*

Yosef Masoudi-Sobhanzadeh[a], Habib Motieghader[a], Ali Masoudi-Nejad[a,*]

[a] Laboratory of system Biology and Bioinformatics, Institute of Biochemistry and Biophysics, university of Tehran, Tehran, iran.

FEATURESELECT, an application for feature selection based on machine learning methods, has been developed in laboratory of system biology and bioinformatics (LBB). FEATURESELECT can be applied on problems needing to select subset of features from given feature set. In continue, we describe some aspects of *FEATURESELECT*.

Implemented language

MATLAB programing language is used for implementing of *FEATURESELECT*. There are some reasons for using it:

1- Because MATLAB is common programing language in different sciences, *FEATURESLECT* has been implemented in t. *FEATURESELECT* can be applied on various areas such as biological data, image processing, handwrite detection, computer science and many other fields.
2- MATLAB is supported by various operating systems such as win, linux, mac.
3- MATLAB is an open source programing language, so everyone can add some new capabilities on *FEATURESELECT*. After investigating new capabilities, we will publish new version of *FEATURESELECT* at https://github.com/yms3786/featureselect.git.

Installation

In order to install *FEATURESELECT*, you must provide some requirements:

1- Install WINSDK.1 in windws or MinGW in linux that include C++ compiler
2- Install MATLAB

After installing the requirements, follow these stages:

1- Copy all files placed in *FEATURESELECT* folder on the one of the available directories.
2- Go to \FEATURESELECT\matlab\ in the intended directory.
3- Click on the one of the matlab files which is available in the entered directory. Notice that the matlab path and the current directory path must be the same.
4- If your application is not working for SVM, write "make" in the matlab's command window and press enter. Be sure that the command successfully completed. In order to get more information about LIBSM, look at https://www.csie.ntu.edu.tw/~cjlin/libsvm/.

Using from FEATURESELECT

After installing the software, you can write "LBBFS" in the matlab command window and use from *FEATURESELECT*. Consider fig.1 and fig.2.

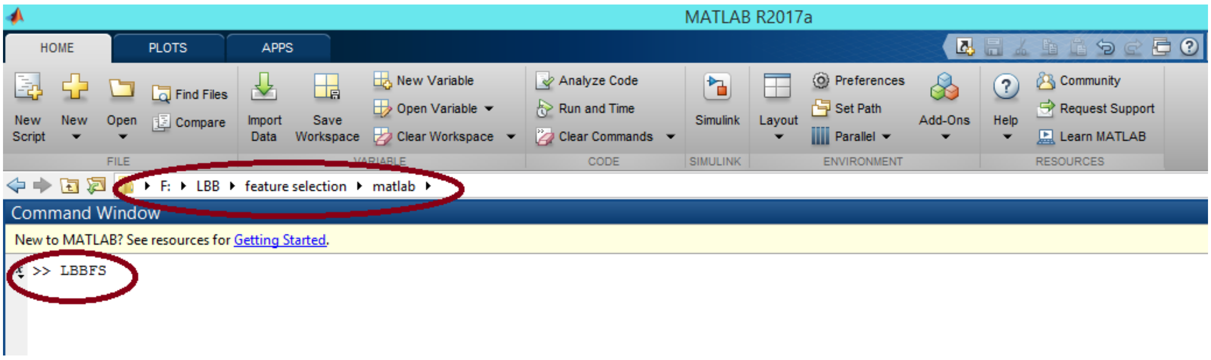

Fig.1: run *FEATURESELECT*

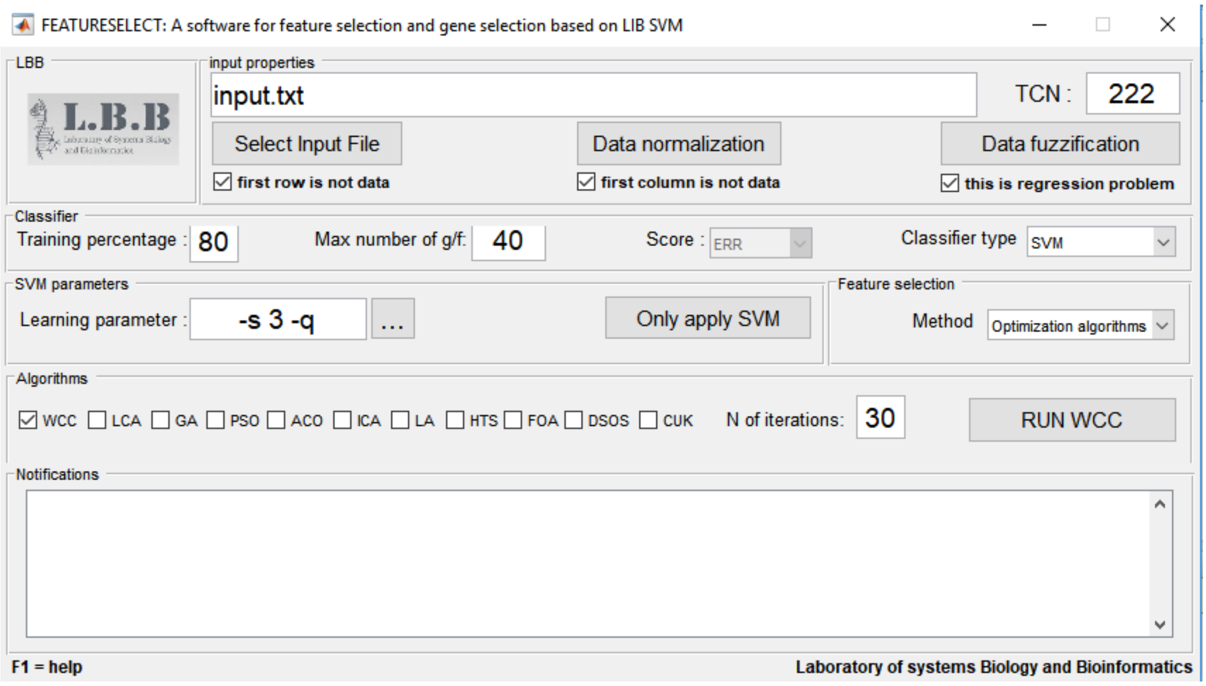

Fig.2: *FEATURESELECT*

Fig.2 shows the *FEATURESELECT* software. This application has several sections:

1- **LBB**: LBB is the ram of our laboratory. This laboratory has been founded by prof ali masoudi-nejad in 2006 at university of Tehran, iran.

2- **Input**: Text, xls and matlab files are acceptable formats of input. You must convert xls file to txt file or m file if it has *struct* structure. TCN is abbreviation for training column number. Your data file and label file must be in the same file. Supposed file name is *input.tx* and the train column number (label of samples) is 222 in it. You can type your input file location in specified box or select it using *select input file button*. For some applications, we need to

normalize or fuzzifize input file. *Data normalization* and *data fuzzification* are designed for this purpose. After clicking on the each of the mentioned buttons, a new file will be created and its name will be added to the specified box (data.txt). When you select an input file, rows of input file arrangement are changed randomly. If first row or first column is not part of input file, click *on first row is not data* or *first column is not data* respectively. *FEATURESELECT* has three main goals: 1- easy using from LIBSVM, ANN and DT, 2- feature selection for regression problems and 3- feature selection for classification problems. The default option is regression. Disable *"this is regression problem"* option if your problem is classification.

3- **Selecting learner type:** Three types of learners are available in *FEATURESELECT*. The first one is SVM. As mentioned before, intended SVM is based on parameters of LIBSVM. The second one is ANN which only includes one parameter (training iteration). We examined some types of artificial neural networks. Finally, the results showed that optimization algorithms can lead to better results in training phase of ANN. Meanwhile, the elapsed time of training phase is enhancing, so it is advised that this type of learner is applied on small datasets. Also, you can select your features by the SVM or DT, and then use ANN in order to obtain an efficient model. The third learner is decision tree (DT). This learner does not need parameter setting.

4- Selecting parameters of LIBSVM: If your learner type is SVM, you can set parameters in this section. Learning parameter which can be selected by the doted button (fig.3) includes LIBSVM's parameters. Training data percentage and maximum number of features which is desirable for your application can be written in the related boxes. Also, if you want to apply LIBSVM on the all of the features (in other words, if you don't want feature selection), click on the *only apply SVM* button**.**

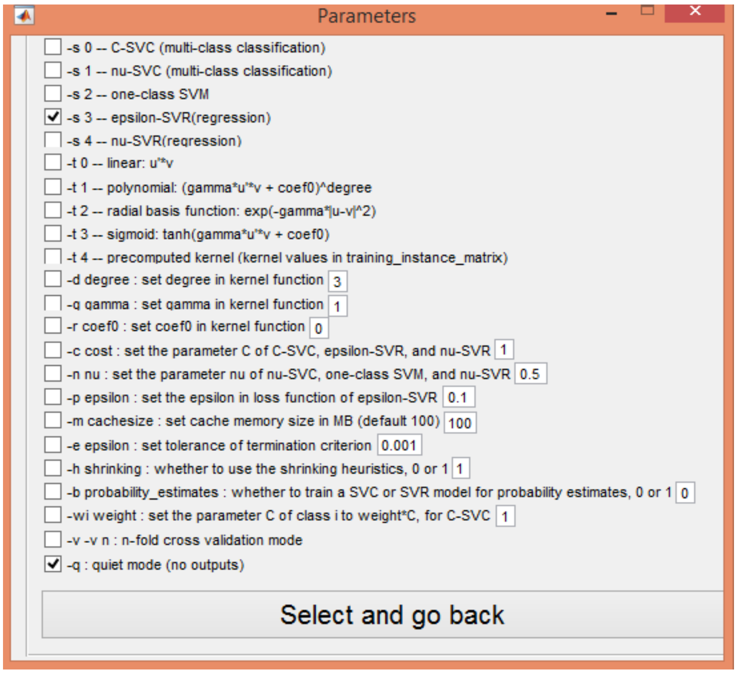

Fig.3: learning parameters of LIBSVM

5- **Feature selection method:** Three types of feature selection methods are available in *FEATURESELECT*: 1- Wrapper method (optimization algorithm). 2- Filter method: this type of feature selection consists of five popular methods. The experimental results show that every learner and every method have their special view relative to dataset, but wrapper methods can lead to better results than filter methods in overall state. 3- Hybrid method: A user can exploit two-step feature selection using combination of the filter and wrapper methods.

6- **Algorithms**: Eleven algorithms have been developed for selecting features from feature set in wrapper method section. It is advised that the optimization algorithms iterated more than 30 times because of stochastic nature of them. You can set number of iterations in the relative box. The new from such as fig.4 which is result of clicking on WCC algorithm will appear. Then you can set the algorithm parameters.

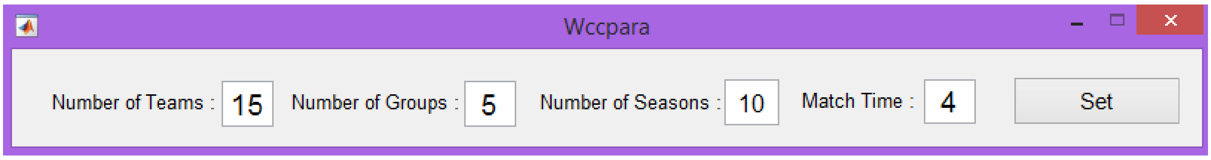

Fig.4: WCC's parameters

The developed algorithms and their reference are:

a) WCC (world competitive contest): this algorithm is inspired by human sport rules. The default values are determined fairly and based on number of LIBSVM calling for all of the algorithms. You can get more information about WCC in http://www.sciencedirect.com/science/article/pii/S2352914816300077.

b) LCA (league championship algorithm): LCA is an algorithm inspired by sport championships. Here is a link for download LCA original paper: http://www.sciencedirect.com/science/article/pii/S1568494613004250.

c) GA (genetic algorithm): GA is the first optimization that mimics natural evolutionary processes. *Crate* and *mrate* are abbreviation for crossover rate and mutation rate in FEATURESELECT. More information about genetic algorithms is available in http://www.sciencedirect.com/science/article/pii/S0377042705000774.

d) PSO (particle swarm optimization): PSO inspired by social behavior of birds. Unlike GA, PSO has not evolutionary operations such as crossover and mutation. Groups of birds fly toward destination. Useful information about PSO is available in http://www.swarmintelligence.org/tutorials.php.

e) ACO (ant colony optimization): this algorithm proposed by marco dorigo in 1992 inspired by ants social behavior. Some aspects of ACO can be found in http://www.sciencedirect.com/science/article/pii/S0167739X0000042X.

f) ICA (imperialist competitive algorithm): atashpaz gargari proposed ICA which is an algorithm inspired by imperialistic competition. You can download relative paper from http://ieeexplore.ieee.org/document/4425083/.

g) LA (learning automata): an automata is an abstract concept. Every cellular automata selects an action from action set and applies it on environment. The selected action will be awarded or penalized. Meybodi published application of LA in https://link.springer.com/chapter/10.1007/3-211-27389-1_35.

h) HTS (heat transfer optimization algorithm): it is a meta-heuristic algorithm which is recently introduced and is based on thermodynamics law. HTS is available in http://www.sciencedirect.com/science/article/pii/S0020025515004764. We showed conduction factor as CDF, convection factor as COF and radiation factor as ROF in FEATURESELECT.
i) FOA (forest optimization algorithm): FOA has been proposed by manizheh ghaemi and has interesting results. This algorithms begins with some randomly created trees as potential solutions. Original research article can be accessed in http://www.sciencedirect.com/science/article/pii/S0031320316300954.
j) DSOS (discrete symbiotic organisms search): DSOS has been published in 2017. It has been showed that DSOS is comparable with the other optimization algorithms, so we implemented it in FEATURESELECT. Original paper of DSOS can be found in http://www.sciencedirect.com/science/article/pii/S0957417417304141.
k) CUK (cuckoo optimization): CUK is proper for continuous nonlinear optimization problem. CUK is inspired by the life of bird family. http://www.sciencedirect.com/science/article/pii/S1568494611001670.

7- Notifications

After running the selected algorithms, the status of program is showed in the notification section.

Outputs

The *results* folder is placed in the directory which contains *FEATURESELECT's* files. For the regression problem, 2 files named *description* and *tbls* are created. For the classification problems, 3 files named *description*, *evaluation* and *tbls* are created. Date and time are added to the end of created file name. The created files contents also are presented in the matlab command window. *Description* file (for both regression and classification problems) includes some information such as number of features and their indices, etc. *Evaluation* file that is specific for classification problems includes statistical measures which are essential for classification problems. For both classification and regression problems, *Tbls* file includes some other statistical information such as p-value, confidence interval, standard deviation, etc. Fig.5 through fig.7 are output instances which have been acquired by batch running of the all algorithms on supposed input file located in *FEATURESELECT* directory.

Command Window

New to MATLAB? See resources for Getting Started.

```
'--------------------------------------------------------------------------------------------'
'This application has been developed in labratoary of system biology and bioinformatics (LBB)'
'--------------------------------------------------------------------------------------------'
'--------------------------------------------------------------------------------------------'
'Algorithm name : WCC'
'Number of selected features :38'
'The selected feature indices are :'
'185,216,169,43,179,106,123,102,141,164,66,146,194,24,129,195,125,36,9,201,191,28,183,11,64,176,196,26,212,31,161,16,45,50,157,114,178,91'
'Elapsed time per one time run based on seconds:24.5241'
'The convergence values of RMSEare:'
'0.21994     0.21994     0.21994     0.21814     0.21814     0.21814     0.21814     0.20654     0.20654     0.20654'
'The average convergence values of RMSEare:'
'0.8632     0.84603     0.75034     0.69892     0.62212     0.60186     0.53374     0.50691     0.50017     0.49957'
'The stability values of RMSEare:'
'0.25986     0.2205     0.28474     0.29193     0.26835     0.27412     0.30341     0.26388     0.25649     0.26002     0.29564     0.2287
'p-value of RMSE:1.2734e-20'
'Confidence interval of RMSE:0.25686     0.2824'
'The value of the test static for error:44.1946'
'The degree of freedom of the test for error:19'
'The estimated population standard deviation for error:0.027284'
'The convergence values of correlation are:'
'0.96536     0.96536     0.96536     0.96511     0.96511     0.96511     0.96511     0.96495     0.96495     0.96495'
'The average convergence values of correlation are:'
```

Fig.5: part of *description* file

1×13 table

AL_NAME	NOF	ET	ER	ER_STD	ER_CI		ER_P	ER_TS	CR	CR_STD	CR_CI	
'WCC'	'38'	'24.5241'	0.20654	0.027284	0.25686	0.2824	1.2734e-20	44.195	0.96495	0.014258	0.92875	0.9421
'LCA'	'34'	'19.6757'	0.22218	0.018497	0.24641	0.26373	2.3672e-23	61.668	0.94586	0.010087	0.92936	0.9388
'GA'	'39'	'2.1273'	0.255	0.02771	0.3032	0.32914	8.4799e-22	51.026	0.92416	0.016478	0.91672	0.93214
'PSO'	'39'	'1.5836'	0.23451	0.032818	0.27529	0.30601	9.9983e-20	39.608	0.91808	0.012044	0.91957	0.93084
'ACO'	'39'	'13.5107'	0.23495	0.051755	0.2838	0.33225	1.6717e-16	26.616	0.93835	0.013881	0.92278	0.93578
'ICA'	'36'	'1.605'	0.24622	0.03733	0.28984	0.32478	3.9402e-19	36.816	0.93262	0.0091037	0.92702	0.93554
'LA'	'39'	'14.0971'	0.18024	0.1082	0.2014	0.30268	2.7134e-09	10.417	0.95058	0.037267	0.91218	0.94706
'HTS'	'39'	'6.2671'	0.31311	0.037151	0.35958	0.39436	7.7401e-21	45.379	0.91462	0.027363	0.89219	0.9178
'FOA'	'19'	'7.9296'	0.1995	0.047763	0.2485	0.29321	4.0893e-16	25.36	0.95832	0.016046	0.92885	0.94387
'DSOS'	'38'	'12.1643'	0.31176	0.038095	0.35486	0.39051	1.5392e-20	43.752	0.9405	0.018199	0.91455	0.93158
'CUK'	'35'	'14.1699'	0.26153	0.023636	0.3006	0.32273	5.5166e-23	58.97	0.95022	0.014646	0.92319	0.9369

Fig.6: part of *tbls* file

EVALUATION =

11×6 table

AL_NAME	SEN	SPC	PRE	FPR	ACC
'WCC'	0.71807	0.71807	0.7195	0.28193	0.72131
'LCA'	0.71537	0.71537	0.72111	0.28463	0.72131
'GA'	0.70292	0.70292	0.70292	0.29708	0.70492
'PSO'	0.70292	0.70292	0.70292	0.29708	0.70492
'ACO'	0.71537	0.71537	0.72111	0.28463	0.72131
'ICA'	0.70455	0.70455	0.75267	0.29545	0.72131
'LA'	0.70725	0.70725	0.73997	0.29275	0.72131
'HTS'	0.67695	0.67695	0.69464	0.32305	0.68852
'FOA'	0.69589	0.69589	0.6989	0.30411	0.68852
'DSOS'	0.70292	0.70292	0.70292	0.29708	0.70492
'CUK'	0.6921	0.6921	0.71646	0.3079	0.70492

Fig.7: part of *evaluation* file (only for classification)

Table.1 shows abbreviation used in *FEATURESELECT* and their complete states.

Table.1: abbreviations

abbreviation	Complete state
ACC	Accuracy
SEN	Sensitivity
SPC	Specificity
FPR	False positive rate
AL_NAME	Algorithm name
PRE	Precision
NOP	Number of features
ET	Elapsed time
ER	Error
CR	Correlation
STD	Standard deviation
CI	Confidence interval
P	p-value
DF	Degree of freedom
ANN	Artificial neural network
DT	Decision tree
NOF	A number of features

Accuracy convergence, accuracy average convergence (accuracy for all of the population in specific generation), accuracy stability, error convergence, error average convergence (for all potential solutions in specific generation) and error stability are plotted for classification problems (fig.8). Error convergence, error average convergence, error stability, correlation convergence, correlation average convergence and correlation stability are plotted for regression problem (dig.9). ROC plot, a statistical measurement that investigates diagnostic ability of classifier, and ROC space are showed in fig.10. You can modify these plots using *view/property editor* menu.

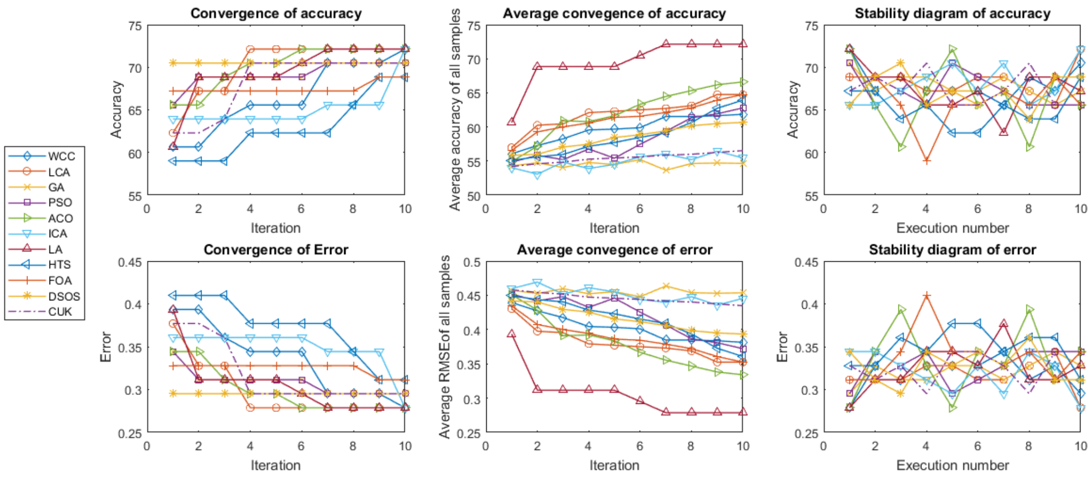

Fig.8: algorithms output for classification problem

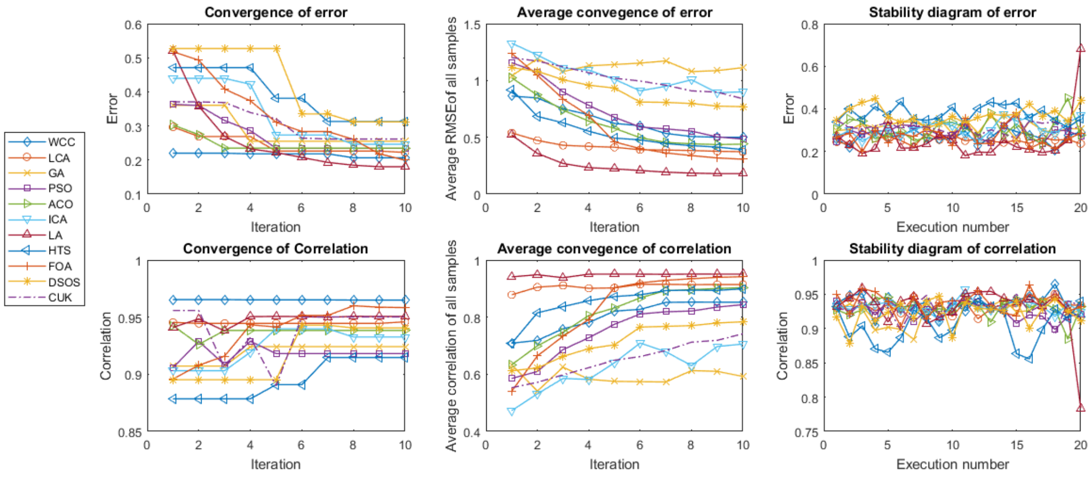

Fig.9: algorithms output for regression problem

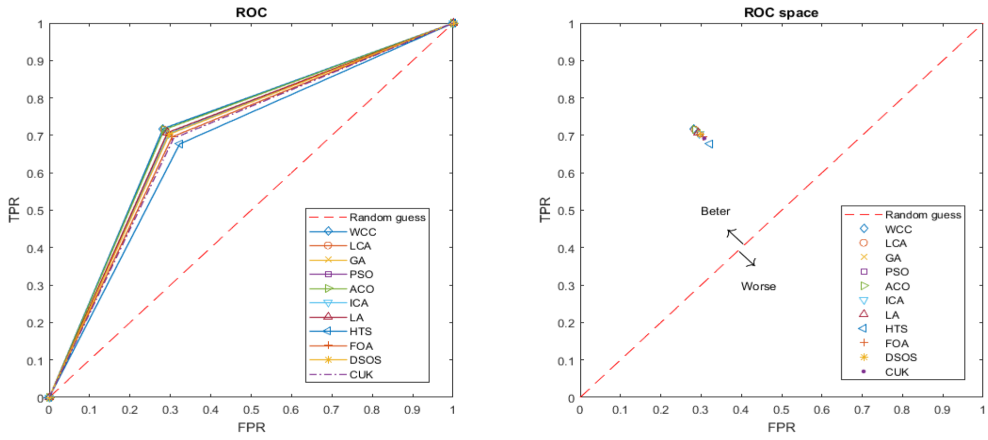

Fig.10: ROC plot and ROC space

In order to exploit hybrid method, a user can follow the bellow steps which are depicted in Figures 11 and 12:

1- Selecting ensemble method:

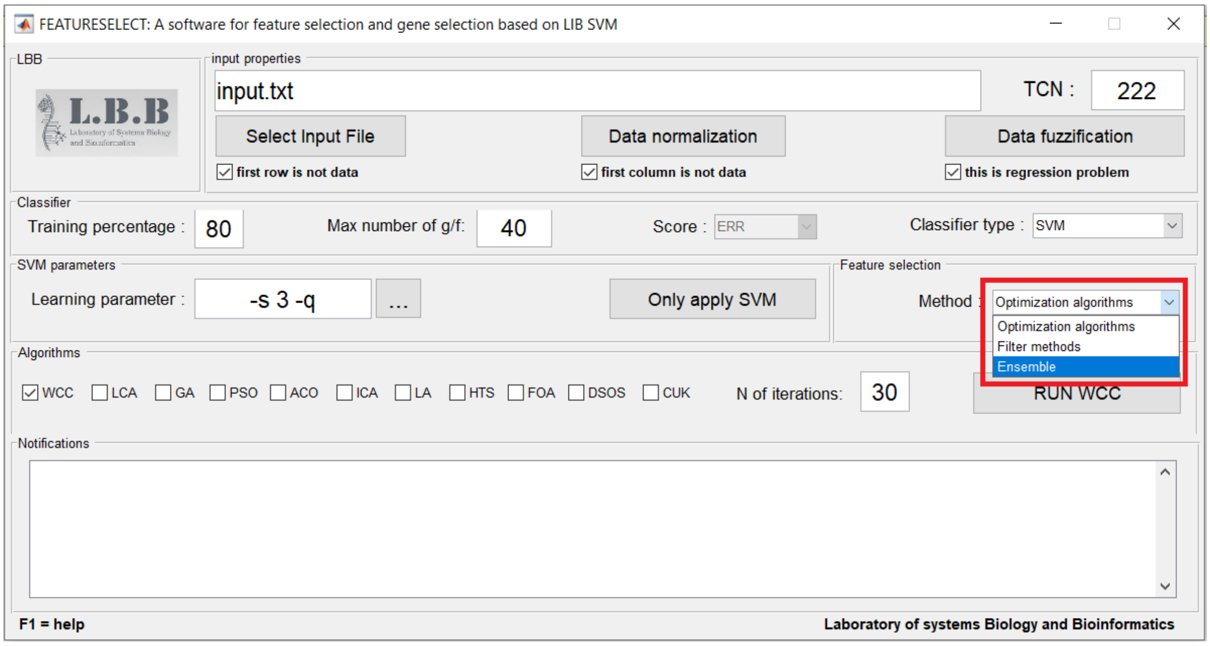

Fig.11: Selecting feature selection method

2- Setting the parameters

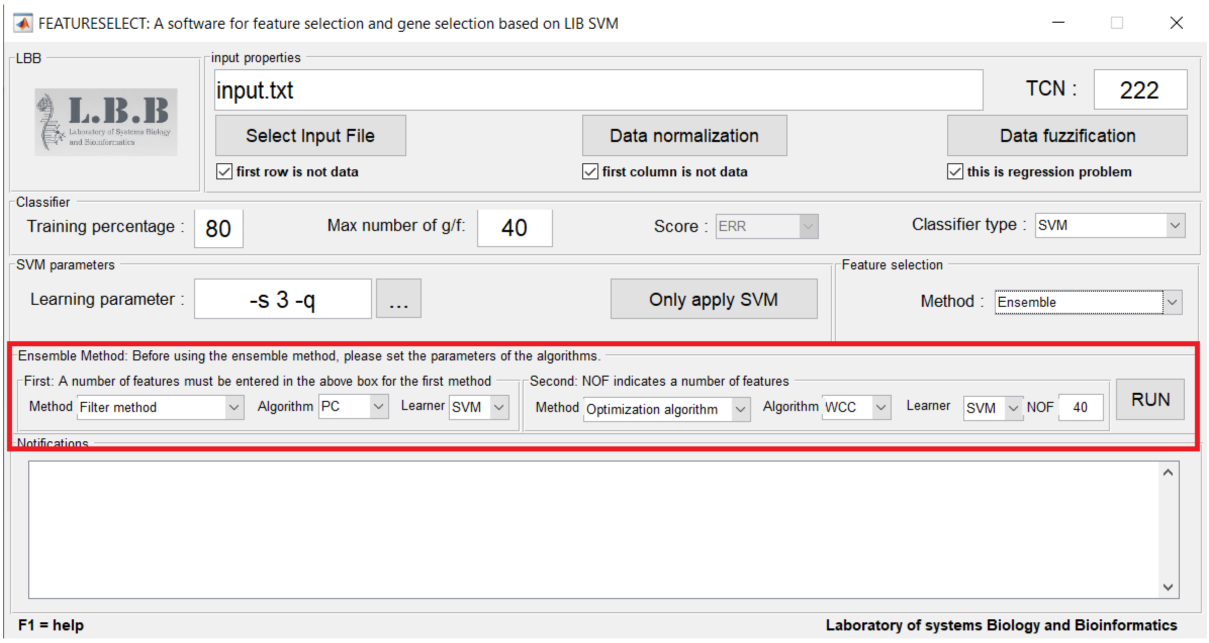

Fig.12: Setting the parameters of the hybrid method
